# *N*-methyl-D-aspartate (NMDA) receptor expression and function is required for early chondrogenesis

**DOI:** 10.1186/s12964-019-0487-3

**Published:** 2019-12-16

**Authors:** Csaba Matta, Tamás Juhász, János Fodor, Tibor Hajdú, Éva Katona, Csilla Szűcs-Somogyi, Roland Takács, Judit Vágó, Tamás Oláh, Ádám Bartók, Zoltan Varga, Gyorgy Panyi, László Csernoch, Róza Zákány

**Affiliations:** 1 Department of Anatomy, Histology and Embryology, Faculty of Medicine, University of Debrecen, Debrecen, Hungary; 2 Department of Physiology, Faculty of Medicine, University of Debrecen, Debrecen, Hungary; 3 Center of Experimental Orthopaedics, Saarland University, Homburg, Germany; 4 Department of Biophysics and Cell Biology, Faculty of Medicine, University of Debrecen, Debrecen, Hungary; 5 Department of Medical Biochemistry, Semmelweis University, Budapest, Hungary

**Keywords:** Chondrogenesis, Chondrocyte, *N*-methyl-D-aspartate receptor, NMDAR, siRNA, Single cell calcium imaging, Glutamate signalling, Glycine

## Abstract

**Background:**

In vitro chondrogenesis depends on the concerted action of numerous signalling pathways, many of which are sensitive to the changes of intracellular Ca^2+^ concentration. *N*-methyl-D-aspartate (NMDA) glutamate receptor is a cation channel with high permeability for Ca^2+^. Whilst there is now accumulating evidence for the expression and function of NMDA receptors in non-neural tissues including mature cartilage and bone, the contribution of glutamate signalling to the regulation of chondrogenesis is yet to be elucidated.

**Methods:**

We studied the role of glutamatergic signalling during the course of in vitro chondrogenesis in high density chondrifying cell cultures using single cell fluorescent calcium imaging, patch clamp, transient gene silencing, and western blotting.

**Results:**

Here we show that key components of the glutamatergic signalling pathways are functional during in vitro chondrogenesis in a primary chicken chondrogenic model system. We also present the full glutamate receptor subunit mRNA and protein expression profile of these cultures. This is the first study to report that NMDA-mediated signalling may act as a key factor in embryonic limb bud-derived chondrogenic cultures as it evokes intracellular Ca^2+^ transients, which are abolished by the GluN2B subunit-specific inhibitor ifenprodil. The function of NMDARs is essential for chondrogenesis as their functional knock-down using either ifenprodil or *GRIN1* siRNA temporarily blocks the differentiation of chondroprogenitor cells. Cartilage formation was fully restored with the re-expression of the GluN1 protein.

**Conclusions:**

We propose a key role for NMDARs during the transition of chondroprogenitor cells to cartilage matrix-producing chondroblasts.

## Background

The poor intrinsic capacity of mature articular cartilage for self-repair is one of the major challenges of regenerative medicine [1]. A better understanding of the early steps of chondrogenesis may enable more efficient hyaline cartilage regeneration. Cartilage formation requires a concerted action of numerous signal transduction molecules, many of which are sensitive to the changes of intracellular Ca^2+^ concentration [2, 3]. We have shown earlier that besides a long-term, day-by-day variation of basal cytosolic Ca^2+^ concentration, differentiating chondrogenic cells in micromass cultures also exhibited rapid, high-frequency oscillatory changes in Ca^2+^ levels, and this phenomenon was sensitive to pharmacological inhibition of voltage-gated potassium channels [4]. More recently, we have also documented the importance of store-operated calcium entry (SOCE) during chondrogenesis [5, 6]. Calcium influx requires the presence and activity of plasma membrane calcium channels, many of which have been reported to be expressed on mature chondrocytes and constitute the ‘chondrocyte channellome’ [7, 8].

The amino acid glutamate is the major excitatory neurotransmitter in the mammalian central nervous system (CNS). It can act on ionotropic (iGluR) and metabotropic (mGluR) receptors. iGluRs are ligand-gated non-selective cation channels, which are classified into alpha-amino-3-hydroxy-5-methyl-4-isoxazolepropionic acid (AMPA), kainate, and *N*-methyl-D-aspartate receptors (NMDARs). Several unique features distinguish NMDARs from both AMPA and kainate receptors, including voltage-sensitive blockade by Mg^2+^, high permeability to Ca^2+^, requirement of glycine as a coagonist, and relatively slow activation and inactivation kinetics. NMDARs are heterotetrameric complexes incorporating two of the ubiquitously expressed GluN1 subunits and two others out of four distinct GluN2 (GluN2A, GluN2B, GluN2C or GluN2D) and/or two GluN3 (GluN3A or GluN3B) subunits [9]. The glutamate binding site of NMDARs is located on the GluN2 subunit [10]. The actual parameters of activation and inactivation kinetics of NMDAR complexes are determined by the specific GluN2 and/or GluN3 subunit composition [11, 12], while the presence of GluN1 is indispensable for the pore formation, assembly and trafficking of the multimer receptor to the cell surface [13].

There is now compelling evidence for glutamate receptor expression and function in non-neuronal tissues, including the pancreas, kidney [14], skeletal system [15], and even in tumours such as melanoma [16]. Glutamate signalling via NMDARs have a complex function in bone remodelling [17] and is involved in the differentiation of osteoblasts and osteoclasts [18]. Mature human articular chondrocytes have also been reported to harbour functional NMDARs [19], possibly involved in mechanotransduction pathways [20]. Cultured rat chondrocytes are also known to express NMDAR subunits [21–23], and NMDARs seem to be related to the altered chondrocyte function detected during osteoarthritis [19]. However, the contribution of glutamate signalling to the regulation of chondrogenesis is yet to be clarified.

In the present work, we aimed at investigating glutamate signalling in chondrogenic cells, focusing on the possible involvement of NMDARs in the regulation of the early steps of in vitro chondrogenesis in a primary chick chondrogenic model system. Here we provide evidence that key components of the glutamatergic signalling pathways are present and functional during in vitro chondrogenesis; we also show the full glutamate receptor subunit mRNA and protein expression profile in chicken chondrogenic cells. Moreover, we prove the involvement of NMDAR function in Ca^2+^ oscillations of chondrogenic cells. In addition, we demonstrate that transient gene silencing of the GluN1 subunit causes a delay in cartilage formation, which is fully restored with the re-expression of the GluN1 protein. On the basis of these observations, we propose a key role for NMDARs during the transition of chondroprogenitor cells to cartilage matrix-producing chondroblasts.

## Methods

### Experimental design

Chicken embryonic limb bud-derived chondroprogenitor cells were used to set up micromass cell cultures, which undergo spontaneous chondrogenic differentiation in vitro. Members of the glutamatergic signalling toolkit were confirmed using PCR and western blotting. The function of the signalling pathway was studied using fluorescent single cell calcium imaging upon NMDAR agonists and antagonists, as well as by transient gene silencing. The effects of the activators and inhibitors of glutamatergic signalling on chondrogenesis were evaluated using histological assessment and by monitoring the expression levels of chondrogenic markers using PCR and western blotting.

### Chicken chondrifying high density cell cultures

Chondrifying micromass cell cultures, established from chondroprogenitor cells isolated from limb buds of early chicken embryos, represent a well reproducible experimental model of cartilage formation [24]. Chondrogenic cells in micromass cultures spontaneously differentiate to matrix-producing chondroblasts on culturing days 2 and 3, and a considerable amount of hyaline cartilage extracellular matrix (ECM) is deposited by day 6 of culturing. Ross hybrid chicken embryos of Hamburger–Hamilton stages 22–24 were used to establish primary micromass cell cultures as previously described [5]. Briefly, distal parts of the limb buds of embryos were removed and droplets of cell suspensions with a density of 1.5 × 10^7^ cells/mL were inoculated into Petri dishes or culture plates (Eppendorf, Hamburg, Germany). Day of inoculation was considered as day 0. Colonies were fed with Ham’s F12 medium (Sigma-Aldrich, St. Louis, MO, USA), supplemented with 10% foetal bovine serum (FBS; Gibco, Gaithersburg, MD, USA), antibiotics/antimicotics (penicillin, 50 U/mL; streptomycin, 50 μg/mL; fungizone, 1.25 μg/mL; TEVA, Debrecen, Hungary), and kept at 37 °C in the presence of 5% CO_2_ in a conventional cell culture incubator. The culture medium was changed on every second day. Cultures were maintained for 6 or 10 days.

### RT-PCR analysis

Cell cultures were dissolved in TRI Reagent (Applied Biosystems, Foster City, CA, USA). After the addition of 20% chloroform samples were centrifuged at 4 °C at 10,000×*g* for 15 min. Samples were incubated in 500 μL of RNase free isopropanol at − 20 °C for 1 h, then total RNA was harvested in RNase-free water and stored at − 80 °C. The assay mixtures for reverse transcriptase reactions contained 2 μg RNA, 0.112 μM oligo(dT), 0.5 mM dNTP, 200 units of High Capacity RT (Applied Bio-Systems) in 1× RT buffer. Primer pairs were designed using the Primer BLAST service and ordered from Integrated DNA Technologies (Coralville, IA, USA). The sequences of primer pairs, the annealing temperatures for each specific primer pair, and the expected amplimer size for each polymerase chain reactions are shown in Additional file 1: Table S1 in the Online Resource. The transcript variants each *GRIN* primer pair may potentially amplify are listed in Additional file 1: Table S2 in the Online Resource.

Amplifications were performed in a programmable thermal cycler (Labnet MultiGene™ 96-well Gradient Thermal Cycler; Labnet International, Edison, NJ, USA) with the following settings: initial denaturation at 94 °C for 1 min, followed by 30 cycles (denaturation at 94 °C, 30 s; annealing at optimized temperatures for each primer pair for 30 s – see Additional file 1: Table S1 in the Online Resource; extension at 72 °C, 30 s) and then final elongation at 72 °C for 5 min. PCR products were analysed by electrophoresis in 1.2% agarose gels containing ethidium bromide.

### Western blot analysis

For western blot analyses, total cell lysates and membrane fractions were used. Total cell lysates for SDS–PAGE were prepared as previously described [25]. For isolation of the membrane fraction, sonicated samples were centrifuged at 50,000×g for 90 min at 4 °C. The resulting pellet was triturated in 50 μL homogenization buffer (50 mM Tris–HCl buffer (pH 7.0), 10 μg/mL Gordox, 10 μg/mL leupeptin, 1 mM phenylmethylsulphonyl fluoride (PMSF), 5 mM benzamidine, 10 μg/mL trypsin inhibitor) supplemented with 1% Triton X-100 at 4 °C. After 1 h of trituration samples were centrifuged again at 50,000×g for 55 min at 4 °C, and the supernatant containing the membrane fraction was used for western blot analyses. Fivefold concentrated electrophoresis sample buffer (20 mM Tris–HCl pH 7.4, 0.01% bromophenol blue dissolved in 10% SDS, 100 mM β-mercaptoethanol) was added to total lysates and membrane fractions to adjust equal protein concentration of samples, and boiled for 5 min.

In each lane, 50 μg of protein was separated by using 7.5% SDS–polyacrylamide gels for western blot analyses. Proteins were then transferred electrophoretically to nitrocellulose membranes. After blocking in 5% non-fat dry milk dissolved in PBS, membranes were exposed to primary antibodies overnight at 4 °C. The details of the primary antibodies applied are summarised in Table 1. Specificity controls for the employed GluN antibodies are shown in Additional file 1: Fig. S1 in the Online Resource. After washing for 30 min in PBST, membranes were incubated with the secondary antibody, anti-rabbit IgG (Bio-Rad Laboratories, CA, USA) in 1:1000 dilution. Membranes were developed and signals were detected using enhanced chemiluminescence (Millipore, Billerica, MA, USA) according to the instructions provided by the manufacturer. Optical density of signals was measured by using ImageJ 1.40 g freeware. For total lysates, loading was controlled by normalizing the results to the optical density values of the loading control (for most of the cases, GAPDH), and then to the untreated (or day 0) cultures. Results of 3 parallel experiments were pooled and presented as bar graphs ± SEM, along with representative membrane images from a single experiment.

**Table 1:** Specifications of primary and secondary antibodies employed for western blotting

Primary antibody	Supplier	Catalogue number	Dilution	Secondary antibody	Supplier	Catalogue number	Dilution
Sox9	Abcam	ab26414 (polyclonal)	1:600	Goat Anti-Rabbit IgG (H + L)-HRP Conjugate	Bio-Rad	1,706,515	1:1000
P-Sox9	Merck	HPA001758 (polyclonal)	1:600	Goat Anti-Rabbit IgG (H + L)-HRP Conjugate	Bio-Rad	1,706,515	1:1000
GluN1	Cell Signaling	4204	1:600	Goat Anti-Rabbit IgG (H + L)-HRP Conjugate	Bio-Rad	1,706,515	1:1000
GluN2A	Abcam	ab14596	1:600	Goat Anti-Rabbit IgG (H + L)-HRP Conjugate	Bio-Rad	1,706,515	1:1000
GluN2B	Cell Signaling	4207	1:600	Goat Anti-Rabbit IgG (H + L)-HRP Conjugate	Bio-Rad	1,706,515	1:1000
GluN3A	Merck/Millipore	07–356	1:200	Goat Anti-Rabbit IgG (H + L)-HRP Conjugate	Bio-Rad	1,706,515	1:1000
GluN3B	Abcam	ab35677	1:200	Goat Anti-Rabbit IgG (H + L)-HRP Conjugate	Bio-Rad	1,706,515	1:1000
GAPDH	Merck/Millipore	AB2302	1:1500	Goat Anti-Rabbit IgG (H + L)-HRP Conjugate	Bio-Rad	1,706,515	1:1000

### Measurement of extracellular glutamate concentration in the culture medium

Concentration of extracellular glutamate released by chondrifying cells into the culture medium was determined by using a Glutamine/Glutamate Determination Kit (Sigma-Aldrich). Measurements were carried out according to the instructions of the manufacturer. 5 droplets of the cell suspension (100 μL each) were inoculated into Petri dishes (Eppendorf), and 4 × 200 μL of culture medium was taken to determine the amount of secreted glutamate on various days of culturing. Background emission was determined by using blanks (both sterile water and Ham’s F12 culture medium). Absorbance was detected at 340 nm using a microplate reader (Chameleon, Hidex Ltd., Turku, Finland). Measurements were performed on 4 parallel samples on each culturing day in 3 independent experiments.

### Measurement of cytosolic free Ca^2+^ concentration and analysis of calcium transients

Measurements were performed on days 1, 2 and 3 on cultures seeded onto 30-mm round coverglasses using the calcium-dependent fluorescent dye Fura-2 as described previously [25]. Fura-2-loaded cells were placed on the stage of an inverted fluorescent microscope (Diaphot, Nikon, Kowasaki, Japan) and viewed using a 40× oil immersion objective. Measurements were carried out in Tyrode’s standard salt solution (containing 1.8 mM Ca^2+^; composition: 137 mM NaCl, 5.4 mM KCl, 0.5 mM MgCl_2_, 1.8 mM CaCl_2_, 11.8 mM HEPES, 1 g/L glucose; pH 7.4) in a perfusion chamber using a dual wavelength monochromator equipment (DeltaScan, Photon Technologies International, Lawrenceville, KY, USA) at room temperature. Excitation wavelength was altered between 340 and 380 nm at 50 Hz and emitted light was detected at 510 nm. Data acquisition frequency was 10 Hz. Ratios of emitted fluorescence intensities (detected at alternating excitation wavelengths; F340/F380) were measured as previously described [25]. Test solutions (20 μM NMDA prepared from Tyrode’s solution) and calcium-free Tyrode’s (containing 5 mM EGTA, without CaCl_2_) were directly applied to the cells through a perfusion capillary tube (Perfusion Pencil™; AutoMate Scientific, San Francisco, CA, USA) with an internal diameter of 250 μm at a 1.5 μL/s flow rate, using a local perfusion system (Valve Bank™ 8 version 2.0, AutoMate Scientific). Ca^2+^ transients were measured in 3 independent experiments.

Spontaneous Ca^2+^ transients were monitored using an LSM 510 META Laser Scanning Confocal Microscope (Zeiss, Oberkochen, Germany) as previously described [5]. All measurements were performed at room temperature. Briefly, cells of 2-day-old micromass cell cultures were incubated for 30 min at 37 °C with 10 μM Fluo-4-AM in Ham’s F12 medium. Calcium imaging was carried out in standard Tyrode’s solution. Test solutions (20 μM NMDA, and 20 μM ifenprodil (Sigma-Aldrich) used as a GluN2B-specific inhibitor) were prepared in Tyrode’s solution. Acquisition of line-scan images started immediately after changing the solution on the cultures. During measurements, only cells exhibiting Ca^2+^ oscillations were investigated, and other cells were disregarded. Line-scan images were acquired at 0.8 ms/line, 512 pixels/line with 7 ms intervals, recording a total of 8192 lines using a 63× water immersion objective. Measurements were carried out in cells from 3 independent experiments. Images were analysed using an automatic event detection software developed in the Department of Physiology of the University of Debrecen, Faculty of Medicine, to determine the FTHM values of the detected spontaneous transients. Statistical comparisons and plotting were performed with Prism version 8.0.1 (Graphpad Software, Inc.). Differences with *P* < 0.05 was considered statistically significant. The box plot diagram shows the individual data points (dots), mean values (+), the minimum and maximum values of the data sets (whiskers), and the 25^th^ and 75^th^ percentiles (left and right borders of the boxes).

### Electrophysiology

For the measurement of ionic currents, standard whole-cell patch-clamp techniques were used on culturing days 2 and 3. Measurements were carried out using Axopatch 200A and 200B amplifiers connected to Axon Digidata 1200 and 1322A data acquisition hardware (Molecular Devices, Sunnyvale, CA, USA). Pipettes were pulled from GC 150 F-15 borosilicate glass resulting in electrodes having 3 to 5 MΩ resistance in the bath solution. Mg^+^-free Na^+^ based bath solution consisted of 145 mM NaCl, 5 mM KCl, 3.5 mM CaCl_2_, 5.5 mM glucose, and 10 mM HEPES, supplemented with 0.1 mg/ml bovine serum albumin (Sigma-Aldrich), pH 7.35. Measured osmolarity of the solution was between 302 and 308 mOsm/L. K^+^ based intracellular solution contained 140 mM KCl, 5 mM NaCl, 2 mM MgCl_2_, 1 mM CaCl_2_, 10 mM HEPES, and 11 mM EGTA; pH 7.22. Osmolarity of the intracellular solution was approximately 295 mOsm/L. Bath perfusion around the measured cell with different test solutions was achieved using a gravity-flow perfusion setup. Current traces were recorded in voltage-clamp mode, while holding the cell membrane at − 60 mV membrane potential or applying 200-ms-long voltage-ramp protocols running from − 120 mV holding potential to + 50 mV every 15 s. To acquire and analyse data, the pClamp9/10 software package (Molecular Devices) was used. Before analysis, current traces were digitally filtered (three-point boxcar smoothing).

### Qualitative and semi-quantitative analysis of metachromatic cartilage matrix production

Cartilage matrix production in 6 or 10-day-old micromass cultures was assessed by the application of the metachromatic dyes dimethyl-methylene blue (DMMB; Aldrich, Germany) or toluidine blue (TB; Reanal, Budapest, Hungary). For the qualitative analysis of cartilage matrix production, micromass cultures established from 30 μL droplets of the cell suspension of different experimental groups were cultured on round coverglasses (Menzel-Gläser, Menzel GmbH, Braunschweig, Germany) in 24-well culture plates. Cultures on days 6 or 10 were fixed and stained with 1% DMMB as described previously [5]. Photomicrographs of metachromatic cartilaginous nodules were taken with a Spot Advanced camera on a Nikon Eclipse E800 microscope (Nikon, Tokyo, Japan). The amount of sulphated matrix components was determined with a semi-quantitative method, by measuring the optical density of extracted TB bound to glycosaminoglycans in mature micromass cultures as described previously [5]. Optical density was measured in samples from 3 cultures of each experimental group in 3 independent experiments.

### Pharmacological modulation of NMDAR function

The following pharmacons were added to the culture medium to modulate NMDAR activity: 20 μM NMDA (Sigma-Aldrich; stock: 20 mM dissolved in sterile water) or 10 μM glycine (Amresco, Solon, OH, USA; stock: 10 mM dissolved in sterile water) were used as agonists; 10 μM DCKA (5,7-dichlorokynurenic acid; Tocris Bioscience, Ellisville, MI, USA; stock: 10 mM dissolved in DMSO) was applied as a competitive antagonist of the glycine-site at the GluN1 subunit; and 20 μM ifenprodil (Sigma-Aldrich; stock: 20 mM dissolved in sterile water) was used for specific inhibition of the GluN2B subunit. NMDA, DCKA and ifenprodil were applied continuously from day 1; glycine was either administered continuously from day 1 or for 2 × 4 h on days 2 and 3 of culturing. The general glycine receptor antagonist strychnine (Sigma-Aldrich; stock: 5 mM dissolved in ethanol) was applied at 5 μM concentration continuously from day 1. Control cultures were treated with appropriate volumes of the respective vehicle (water, ethanol or DMSO).

### Measurement of cell proliferation and mitochondrial activity (cell viability)

Rate of cell proliferation was determined by monitoring the amount of incorporated radioactivity from ^3^H-thymidine. Culture medium containing 1 μCi/mL ^3^H-thymidine (diluted from methyl-^3^H-thymidine; 185 GBq/mM, Amersham Biosciences, Budapest, Hungary) was added to cells seeded into 96-well assay plates (Wallac, PerkinElmer Life and Analytical Sciences, Shelton, CT, USA) for 16 h on day 3 after the application of NMDAR agonists or antagonists, or on day 10. After washing with PBS, proteins were precipitated with ice-cold 5% trichloroacetic acid, and rinsed with PBS again. Colonies were then air-dried for 1 week and radioactivity was counted by a liquid scintillation counter (Chameleon, Hidex). Measurements were carried out in 10 samples of each experimental group in 5 independent experiments. Scintillation counting data of the experimental groups have been normalised to those of the respective vehicle controls and shown as percentage increases or decreases.

For mitochondrial activity (cell viability) assays, cells cultured in 96-well plates were used. 10 μL MTT reagent (3-[4,5-dimethylthiazolyl-2]-2,5-diphenyltetrazolium bromide; 5 mg MTT/1 mL PBS) was pipetted into each well. Cells were incubated for 2 h at 37 °C, and following the addition of 500 μL MTT solubilizing solution, optical density was measured at 570 nm (Chameleon, Hidex). Optical density readings of the experimental groups have been normalised to those of the respective vehicle controls and shown as percentage increases or decreases.

### Transient transfection with GRIN1 siRNA

The mRNA sequence of the chicken NMDAR GluN1 subunit (*GRIN1*) was downloaded from GenBank (accession number: AY510024) and a specific siRNA construct (SMARTpool siRNA kit, Dharmacon, Inc., CO, Lafayette, USA) was designed for transient gene silencing. The Custom siRNA SMARTpool consisted of 4 individual siRNAs with the following sequences; Oligo ID: FARED 000005, 5′–ACA GGA AGU UUG CCA AUU AUU–3′; Oligo ID: FARED 000007, 5′–CCA AGU ACU CGG AGG GUG UUU–3′; Oligo ID: FARED 000009, 5′–GAU CGU AAC AAU UCA UCA AUU–3′; Oligo ID: FARED 000011, 5′–CCA CAU AAG UGA UGC AGU GUU–3′. As a specificity control, a non-targeting pool (Cat. No.: D-001810-10-05) was used. The DharmaFect delivery system protocol was performed on the freshly isolated cell suspension with a density of 1.5 × 10^7^ cells/mL (on day 0 of culturing). 100 or 30 μL mixtures of transfection reagent and cell suspension were inoculated into Petri dishes or 24-well plates, respectively. After 2 h, colonies were fed with Ham’s F12 medium supplemented with 10% FBS. mRNA expression and rate of proliferation were investigated after 48 h, whereas protein expression and mitochondrial activity of cells were measured after 3 and 10 days post-transfection. Control cultures were treated with non-targeting (NT) siRNA and equal volumes of the transfection reagent.

### Immunocytochemistry

Cells of different experimental groups were cultured on coverslips as described previously. Cells were fixed in Saint-Marie’s fixative (99% ethanol and 1% anhydrous acetic acid) for 1 h. After washing in 70% ethanol, non-specific binding sites were blocked in PBST supplemented with 1% bovine serum albumin (BSA, Amresco LLC, Solon, OH, USA) at 37 °C.

For immuncytochemical staining, cells were incubated with anti-Sox9 (Abcam, Cambridge, UK; Cat. No.: ab26414) at a dilution of 1:600, and anti-GluN1 (Cell Signaling, Danvers, MA, USA; Cat. No.: 5704) antibodies at a dilution of 1:500 at 4 °C overnight. Primary antibodies were visualised with anti-rabbit Alexa Fluor 555 or anti-mouse Alexa Fluor 488 secondary antibodies (Life Technologies Corporation, Carlsbad, CA, USA; Cat. No.: A27039; A28175) at a dilution of 1:1000. Cultures were mounted in Vectashield Hard Set mounting medium (Vector Laboratories Ltd.) containing DAPI to visualise the nuclei of cells. For investigation of subcellular localization of Sox9 and GluN1, images were taken with an Olympus FV1000S confocal microscope (Olympus Co. Tokyo, Japan) using a 60× oil immersion objective (NA: 1.3). For excitation, laser lines of 543 nm and 488 nm were used. The average pixel time was 4 μs. Images of Alexa Fluor 555, Alexa Fluor 488 and DAPI were overlaid using Adobe Photoshop version 10.0 software.

### Statistical analysis

All data are representative of at least three different experiments. Averages are expressed as mean ± SEM and statistical analysis was performed using Student’s unpaired two-tailed *t*-test followed by Dunnett’s multiple comparison test (*P < 0.05). Differences in the distribution of the length of spontaneous events following NMDA challenge were analysed using a chi-squared test.

## Results

### Chondrogenic mesenchymal cells express NMDAR subunits and secrete glutamate

**Fig. 1:**
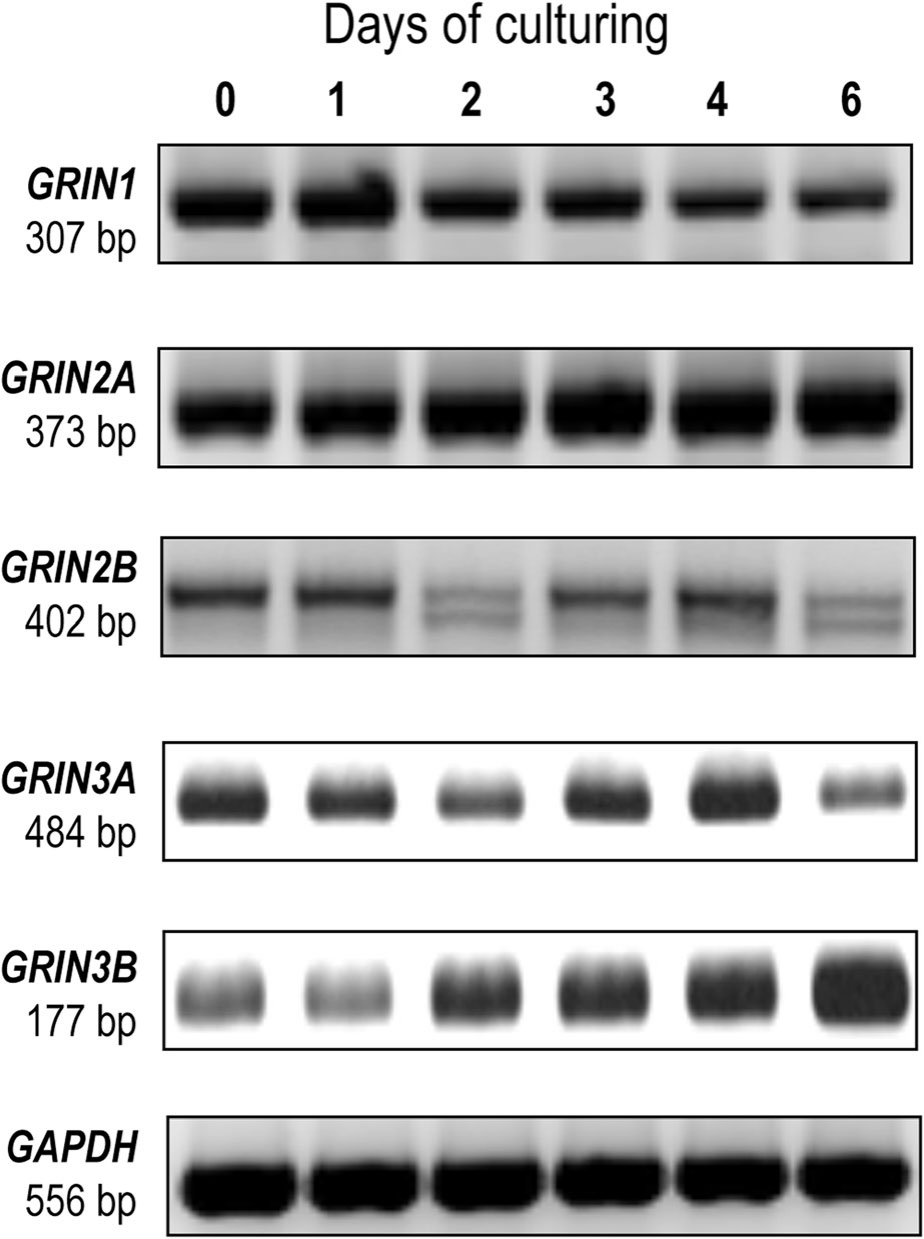
NMDAR subunit expression profile of chondrifying chicken micromass cultures during the entire culturing period (days 0–6). mRNA expression profiles of *GRIN1*, *GRIN2A*, *GRIN2B*, *GRIN3A*, *GRIN3B* and *GRIN3C* (*n* = 3 for each culturing day). Assays for *GRIN2C* and *GRIN2D* have also been performed but no bands at the expected size were detected (see Additional file 1: Fig. S2 in the Online Resource)

**Fig. 2:**
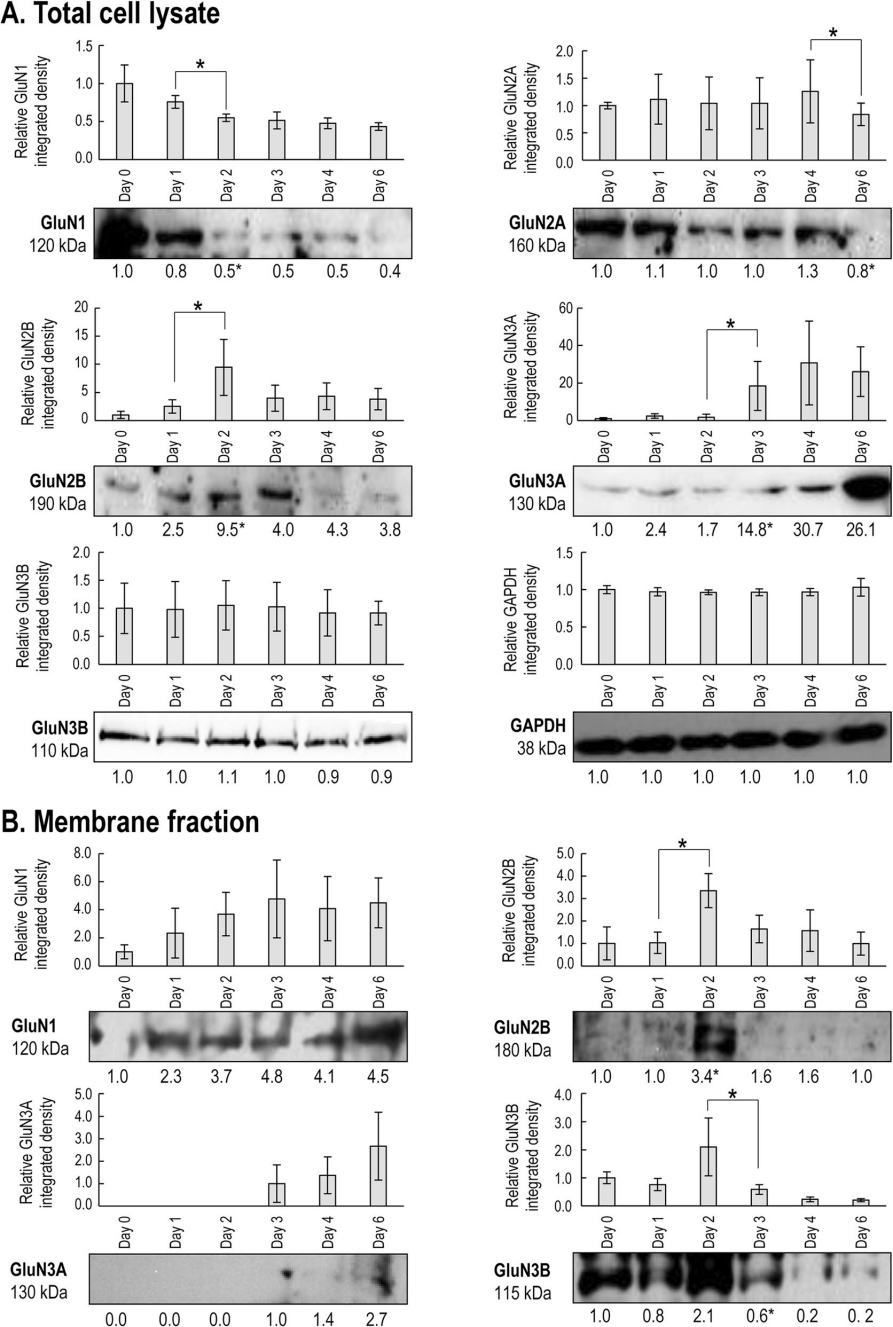
NMDAR subunit expression profile of chondrifying chicken micromass cultures during the entire culturing period (days 0–6). **a**. Protein expression profiles of GluN1, GluN2A, GluN2B, GluN3A and GluN3B subunits in total cell lysates (*n* = 3 for each culturing day). **b**. Protein expression profiles of GluN1, GluN2B, GluN3A and GluN3B subunits in the membrane fraction (*n* = 3 for each culturing day). Immunoblotting for GluN2A in the membrane fraction has also been performed but no bands at the expected size were detected (see Additional file 1: Fig. S2 in the Online Resource). Optical densities of bands from 3 parallel experiments were pooled and presented as bar graphs ± SEM, along with representative membrane images from a single experiment. The representative membrane images are out of 3 independent experiments, each showing a similar expression profile. For the total lysates, data normalization was performed by normalizing each data to the optical density values of the loading control (GAPDH) on the same day, and then to the day 0 cultures. For the membrane fraction, optical density values were normalized to the day 0 cultures. Significant (*P < 0.05) changes in protein levels of the mean optical densities of each day from 3 experiments relative to the previous day (mean) are marked

mRNA expressions of the genes coding for GluN1, GluN2A, GluN2B, GluN3A and GluN3B subunits, but not those of GluN2C or GluN2D subunits, were detected in chondrifying micromass cultures by PCR reactions on various days of culturing (Fig. 1; see also Additional file 1: Fig. S2 in the Online Resource). We observed the protein-level expression profiles of the same subset of NMDAR subunits in total cell lysates using western blotting (Fig. 2a). The protein expression of the GluN1 subunit showed a gradual, significant decline during chondrogenesis; signals for GluN2A were constant with a significant decrease in mature micromass cultures; and a peak for GluN2B subunit expression was observed on days 2 and 3, when ECM-producing chondroblasts first appear in micromass cultures. GluN3A protein was giving very strong signals from culturing day 3, while GluN3B was present throughout the entire culturing period at a steady level.

Membrane fractions were also prepared from micromass cultures and were probed with the same antibodies as the total lysate. The bands that appeared in these blots represented those fractions of the GluN subunit pool that have actually been translocated to the membrane. In the membrane fraction, protein expressions of the GluN1, GluN2B and GluN3B subunits were detected only. GluN1 expression in the membrane fraction was showing an upward trend; GluN2B and GluN3B showed significantly stronger signals on day 2; and GluN3A could only be detected in the membrane at the end of the 6-day-long culturing period. However, no membrane signals were observed for the GluN2A subunit (Fig. 2b; see also Additional file 1: Fig. S2 in the Online Resource), suggesting that although present in the cytoplasm, GluN2A was not translocated to the plasma membrane (and is therefore probably not active). The expression of the above subunits has also been confirmed using immunocytochemistry (Additional file 1: Fig. S3 in the Online Resource). Besides NMDAR subunits, ionotropic (AMPA and kainate) and metabotropic glutamate receptor subunit, as well as glycine receptor subunit mRNA expression profiles were also screened in micromass cultures during chondrogenesis (Additional file 1: Fig. S4 in the Online Resource).

Having confirmed the expression of various NMDAR subunits at the protein level, we aimed at determining whether chondrifying cells of micromass cultures secreted glutamate, the natural agonist of NMDARs, into the culture medium. We found higher concentrations of glutamate in the supernatants of micromass cultures than in cell-free media on each day of culturing, suggesting the spontaneous release of endogenous glutamate from the cells. The concentration of secreted glutamate was in the range of 2–11 nmol/mL of medium, as revealed by the colorimetric glutamine/glutamate determination assay (Additional file 1: Fig. S5 in the Online Resource). mRNA and protein expressions of the vesicular glutamate transporters 1 and 2 (VGLUT1 and VGLUT2) were also detected (Additional file 1: Fig. S6 in the Online Resource).

### Local application of NMDA evoked Ca^2+^ transients during single cell measurements

**Fig. 3:**
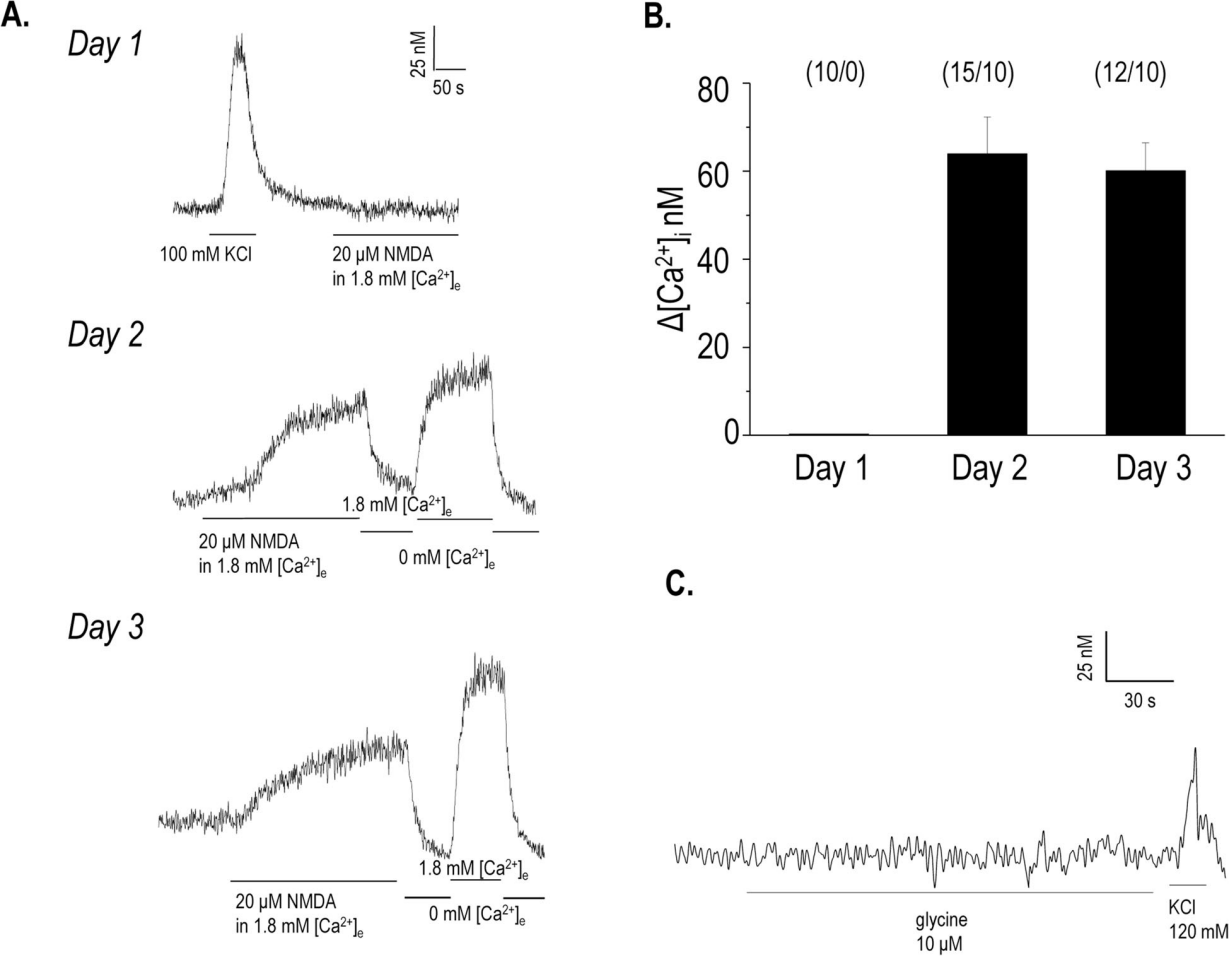
Effect of locally administered 20 μM NMDA on the cytosolic Ca^2+^ levels in Fura-2-loaded cells in micromass cultures at room temperature. **a**. Ca^2+^ transients evoked by administration of NMDA in cells on different days of culturing. When not indicated, Ca^2+^ transients were measured in the presence of 1.8 mM [Ca^2+^]_e_. Representative records of 3 independent experiments. Lines under graphs indicate the application of NMDA, KCl (120 mM) or Ca^2+^-free Tyrode’s. **b**. Changes in the peak amplitude of NMDA-evoked Ca^2+^ transients (average ± SEM) detected on different days of culturing in Fura-2-loaded cells. Numbers above bars represent the ratio of cells responding to the NMDA challenge. Representative data of 3 independent experiments, each showing similar trends of changes. **c**. Record showing the lack of Ca^2+^ transients evoked by administration of 10 μM glycine measured in the presence of 1.8 mM [Ca^2+^]_e_. Representative record of 3 independent experiments

Next, we wanted to study the effects of NMDAR signalling on the calcium homeostasis of differentiating chondrocytes. Calcium transients were recorded on different days of culturing following the local application of 20 μM NMDA. Chondroprogenitor cells did not respond to challenges of NMDA on day 1 of culturing (Fig. 3a). This, however, was not due to their inability to increase their cytosolic Ca^2+^ concentration ([Ca^2+^]_i_) since a depolarization evoked by the addition of 100 mM KCl to the external medium resulted in a rapid rise in [Ca^2+^]_i_. On the other hand, starting from day 2 of culturing, differentiating chondrocytes of micromass cultures generated a pronounced elevation of [Ca^2+^]_i_ upon the application of NMDA (Fig. 3a). The response was slow to develop and was clearly dependent on the presence of external calcium, since the removal of [Ca^2+^]_e_ brought [Ca^2+^]_i_ back to its resting value, while re-administration of 1.8 mM [Ca^2+^]_e_ again increased [Ca^2+^]_i_. This phenomenon was not attributable to aspecific increases in Fura-2 fluorescence over time (Additional file 1: Fig. S7 in the Online Resource). This indicated that in addition to NMDARs, local application of NMDA might have opened and/or influenced other influx pathways for calcium in the plasma membrane that were slow to activate and also slow to inactivate. The average amplitude of the Ca^2+^-peaks following the NMDA challenge was 60 nM on both days 2 and 3 (Fig. 3b).

Although glycine is absolutely required to activate the Gly-binding site on the GluN1 subunit as a co-agonist and is also an agonist of NMDARs containing GluN3 subunits [26], local application of 5 μM glycine alone had no influence on [Ca^2+^]_i_ on any culturing days (Fig. 3c).

**Fig. 4:**
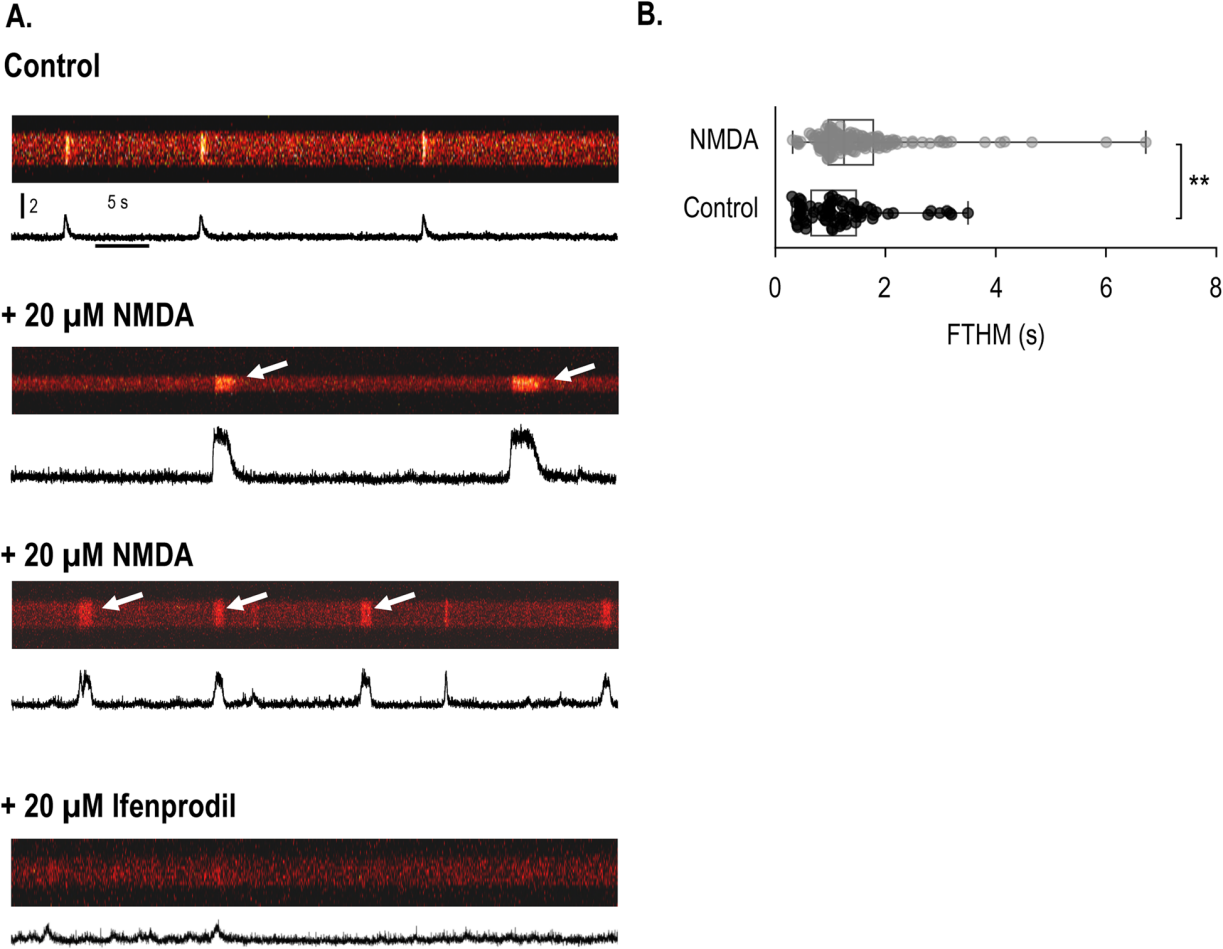
Spontaneous Ca^2+^ oscillations in cells of micromass cultures on day 2 of culturing. Prior to measurements, cells were loaded with Fluo-4-AM for 30 min. Ca^2+^ oscillations were observed without agonist stimulation in Tyrode’s solution containing 1.8 mM [Ca^2+^]_e_ at room temperature. Acquisition of line-scan images started immediately after changing the bath solution on cultures. **a**. Spontaneous Ca^2+^ oscillations in normal ([Ca^2+^]_e_ = 1.8 mM) Tyrode’s solution, or following application of 20 μM NMDA or 20 μM ifenprodil. Line-scan diagrams show representative data out of 3 independent experiments. **b**. Box plot showing the distribution of the length of spontaneous events; control cells are black, NMDA-treated cells are grey. The box plot shows the full time at half maximum (FTHM) values of the detected spontaneous transients (dots: individual data points; +: mean values; whiskers: minimum and maximum values of the data sets; left and right borders of the boxes: the 25^th^ and 75^th^ percentiles). A significant (**P < 0.01, with Mann-Whitney test) change in event length following NMDA challenge relative to the control was detected

To explore whether NMDAR signalling was associated with spontaneous Ca^2+^ oscillations reported earlier in this model system [5], we applied 20 μM NMDA and the GluN2B-specific inhibitor ifenprodil (20 μM) during confocal Ca^2+^ measurements. Administration of NMDA altered the length of the spontaneous Ca^2+^ events (Fig. 4a, arrows); the frequency of very short (0.5 s) events reduced, and at the same time, the frequency of longer (1.5–2.5 s) events increased compared to the control (*P* = 0.0002), indicating a similar mechanism to what we have seen during single cell measurements (Fig. 4b). The function of NMDARs (or the conductance(s) activated by the external NMDA challenge) seemed to be required for spontaneous Ca^2+^ events as the GluN2B-specific channel inhibitor ifenprodil completely abolished the Ca^2+^ oscillations.

To test the ionic flow across the plasma membrane in differentiating chondrocytes on culturing days 2 and 3 following NMDA challenge, whole-cell patch-clamp measurements were carried out in voltage-clamp mode. Current traces were recorded while applying a holding potential of − 60 mV. Application of 20 μM NMDA and 5 μM glycine or 300 μM NMDA and 10 μM glycine simultaneously in the extracellular solution had no significant effect on the amplitude of the measured current when the cell was held continuously at − 60 mV (Additional file 1: Fig. S8A–C in the Online Resource). To test the possible effect of the agonists, voltage ramp protocols were also applied with which any change of the reversal potential due to the activation of NMDARs could be detected. Application of the test substances, however, had no significant effect on the reversal potential of the measured current (Additional file 1: Fig. S8D–F in the Online Resource).

### Pharmacological modulation of NMDARs exerted inconsistent effects on cartilage formation

**Fig. 5:**
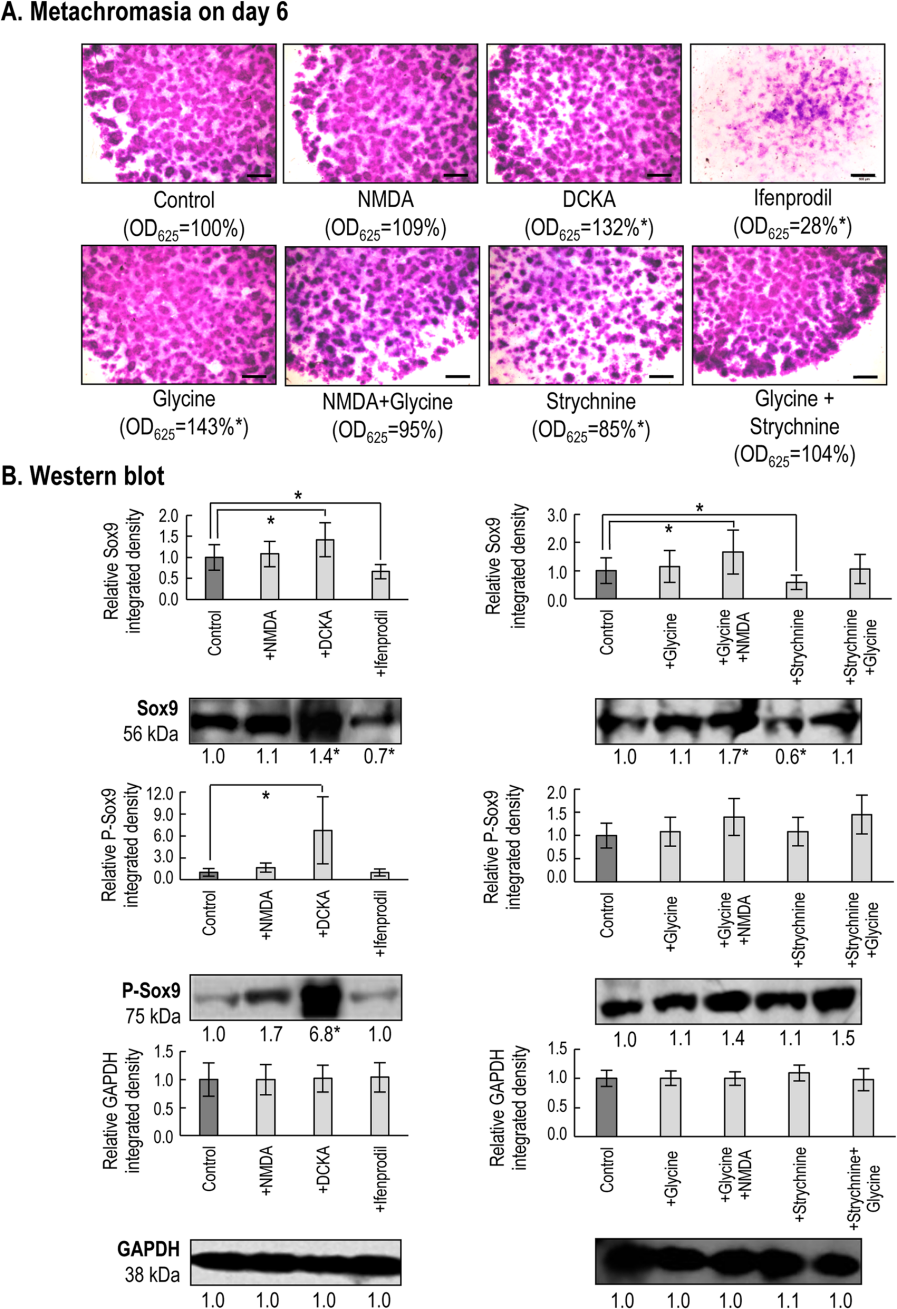
Effects of pharmacological modulation of NMDAR function on chondrogenesis. **a**. Metachromatic cartilage areas in 6-day-old high density colonies were visualised with DMMB dissolved in 3% acetic acid (pH 1.8). Metachromatic (purple) structures represent cartilaginous nodules formed by multiple cells and an ECM rich in polyanionic GAGs (*n* = 3 for each experimental condition). Original magnification was 2×. Scale bar, 1 mm. Optical density (OD_625_) was determined in supernatants of 6-day-old cultures (*n* = 3 for each experimental condition) containing toluidine blue extracted with 8% HCl dissolved in absolute ethanol. Representative data out of 3 independent experiments. Statistically significant (*P < 0.05) differences in extinction (OD_625_) of samples for TB relative to the respective vehicle control are marked. **b**. Protein expression and phosphorylation status of the master chondrogenic transcription factor Sox9 in 3-day-old cultures (*n* = 3 for each experimental condition). Optical densities of bands from 3 experiments were pooled and presented as bar graphs ± SEM, along with representative membrane images from a single experiment. The representative membrane images are out of 3 independent experiments, each showing a similar expression profile. Data normalization was performed by normalizing each data to the optical density values of the loading control (GAPDH) for the same experimental condition, and then to the control cultures (dark grey columns). Significant (*P < 0.05) alterations in protein levels of the mean signal densities from 3 experiments relative to the respective controls (mean) are marked

When 20 μM NMDA was applied continuously to chondrogenic cultures from the first day of culturing, metachromatic ECM formation remained unchanged by day 6 compared to the control (Fig. 5a). When applied from culturing day 1, DCKA, the competitive antagonist of the Gly-site on the GluN1 subunit, caused a significant elevation in metachromatic cartilage matrix production. On the contrary, the GluN2B-specific antagonist ifenprodil (20 μM) almost completely blocked cartilage formation (Fig. 5a).

Since NMDA alone did not affect chondrogenesis, we also administered glycine (at 10 μM) from culturing day 1 either alone or in combination with NMDA. Whilst glycine alone significantly enhanced metachromatic cartilage matrix production, when NMDA and glycine were applied together (i.e. to achieve full activation of NMDARs) they failed to alter the amount of cartilage ECM produced by day 6 compared to the control (Fig. 5a). To check whether glycine was acting on glycine receptors in addition to (or instead of) NMDARs, we applied 5 μM strychnine during further experiments. Strychnine alone reduced the amount of cartilage ECM production, while co-application with glycine restored cartilage formation (Fig. 5a).

The protein levels and the phosphorylation status of Sox9, the master transcription factor of chondrogenesis, also followed the changes observed in metachromatic cartilage matrix formation (Fig. 5b) as determined by western blotting. Intriguingly, the only significant increase in P-Sox9 levels was caused by DCKA.

**Fig. 6:**
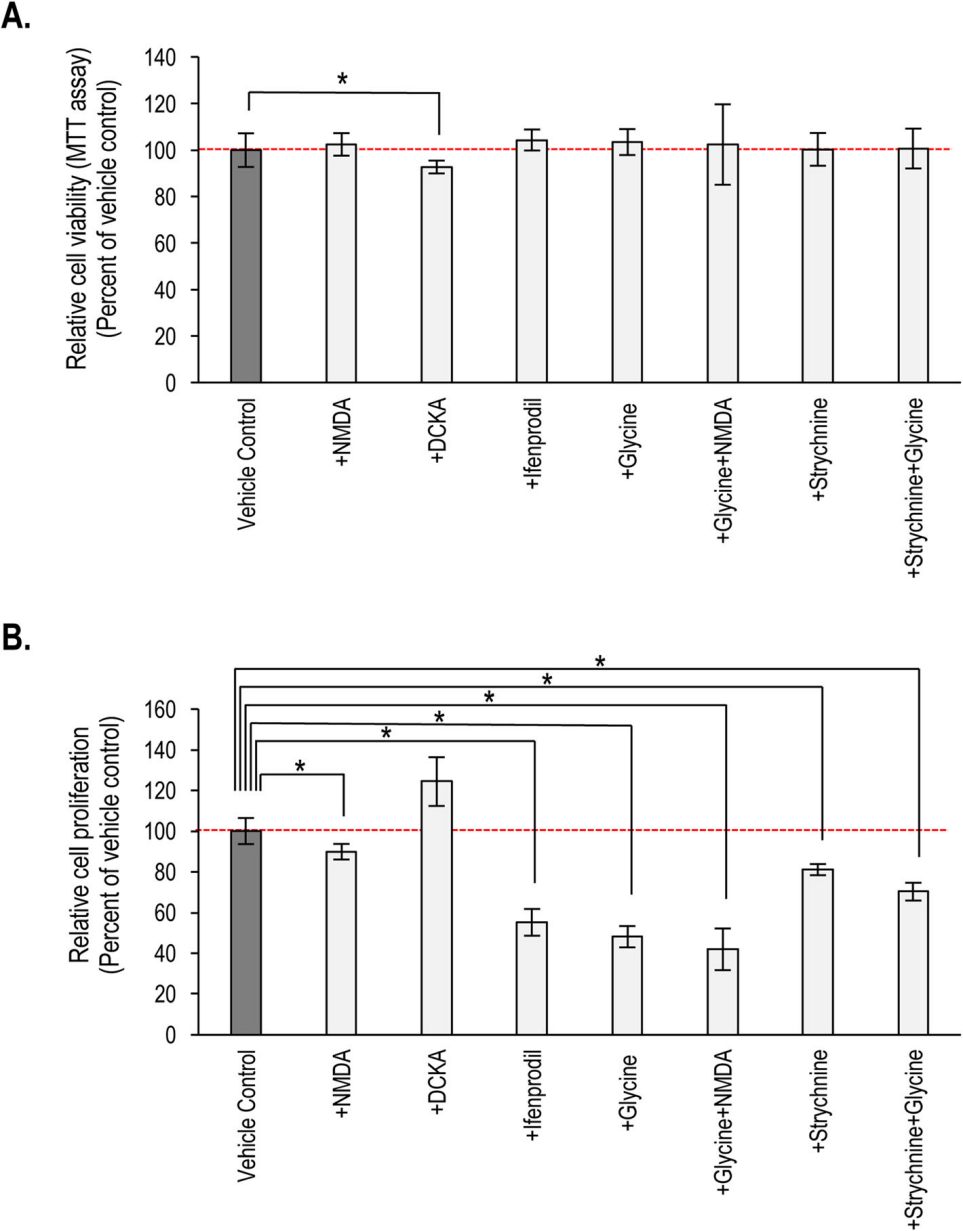
Mitochondrial activity, a measure of cell viability (**a**) and rate of proliferation (**b**) on day 3 determined by MTT test and ^3^H-thymidine incorporation assays, respectively, in micromass cultures (*n* = 3 for each experimental condition) following pharmacological modulation of NMDAR function. Representative data (average ± SEM) out of 3 independent experiments showing the same tendency of changes. For each experimental group, data were normalized to that of the respective vehicle control (not shown individually). Statistically significant (*P < 0.05) differences compared to the vehicle control are marked

As a high cellular density established via rapid proliferation and the appropriately high number of viable cells are a prerequisite to in vitro chondrogenesis, these parameters were also monitored. Except for DCKA, none of the other applied pharmacons caused significant alterations in mitochondrial activity (a parameter of cellular viability) as detected by MTT assay (Fig. 6). However, proliferation rate was significantly reduced by the GluN2B subunit inhibitor ifenprodil, and the GluN1 subunit coagonist and GluN3 agonist glycine (either alone or co-applied with NMDA, with or without the GlyR inhibitor strychnine). On the contrary, proliferation rate was found to be significantly elevated by DCKA. The glycine receptor blocker strychnine also significantly reduced the proliferation rate in 3-day-old micromass cultures (Fig. 6).

### Transient gene silencing of GluN1 subunit delays in vitro chondrogenesis

**Fig. 7:**
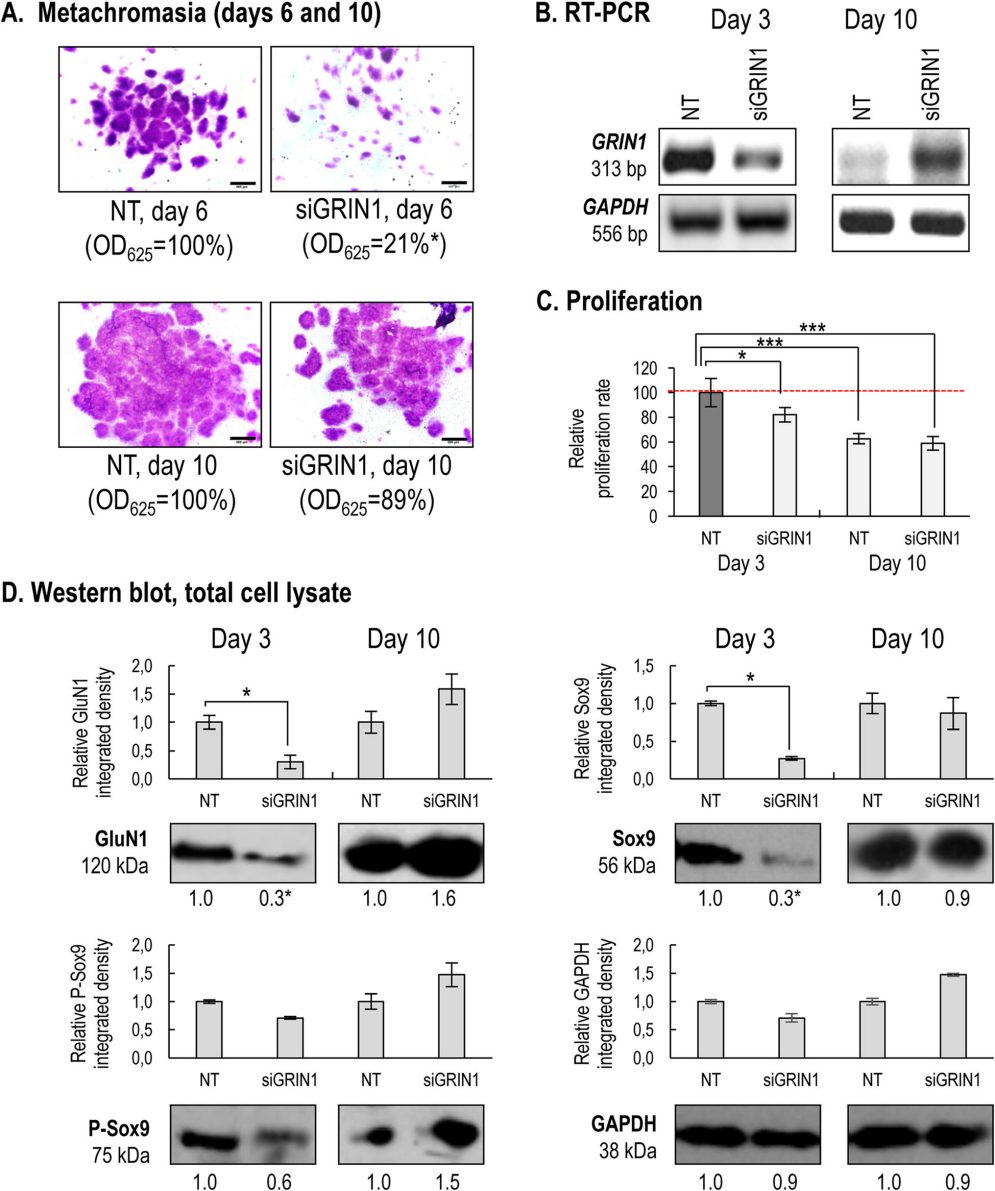
Effects of transient gene silencing using of the *GRIN1* siRNA on chondrogenesis. **a**. Metachromatic cartilage areas in 6 or 10-day-old high density colonies (*n* = 3 for each experimental condition on each day) were visualised with DMMB. Original magnification was 2×. Scale bar, 1 mm. Optical density (OD_625_) was determined in supernatants of 6 or 10-day-old cultures (*n* = 3 for each experimental condition on each day) containing extracted toluidine blue. Statistically significant (*P < 0.05) differences in extinction (OD_625_) of samples for TB are marked. **b**. mRNA expression of the *GRIN1* gene following gene silencing with siRNA on culturing days 3 and 10 (*n* = 3 for each experimental condition on each day). Non-targeting (NT) siRNA was used as a control, and *GAPDH* was used as a reference gene. **c**. Proliferation rate on days 3 and 10 in micromass cultures (*n* = 3 for each experimental condition on each day; average ± SEM). Significant (*P < 0.05; ***P < 0.0001) changes of proliferation rate relative to the non-targeting (NT) control (on day 3) are marked. **d**. Protein expression of the GluN1 subunit, as well as expression and phosphorylation status of Sox9 in 3- and 10-day-old cultures (*n* = 3 for each experimental condition on each day). GAPDH was used as an internal control. Optical densities of bands from 3 experiments were pooled and presented as bar graphs ± SEM, along with representative membrane images from a single experiment. The representative membrane images are out of 3 independent experiments, each showing a similar expression profile. Data normalization was performed by normalizing the density date of the gene silenced cultures to the optical density values of the loading control (GAPDH) for the same experimental condition, and then to the NT control cultures (dark grey columns). Significant (*P < 0.05) alterations in protein levels of the mean signal densities from 3 experiments relative to the NT control (mean) on the same culturing day are marked. All panels show representative data out of 3 independent experiments showing the same tendency of changes

Considering that NMDARs are not functional without GluN1 subunits [13], we aimed to downregulate its mRNA expression level by transient gene silencing during the earliest stage of chondrogenesis. *GRIN1* siRNA-encoding vector was introduced into freshly isolated chondrogenic cells (on day 0). *GRIN1* silencing had a profound impact on chondrogenesis: metachromatic ECM production decreased to approximately 20% of non-targeting control cultures (NT), as demonstrated by staining with DMMB and TB on day 6 of culturing (Fig. 7a). The mRNA level of *GRIN1* was reduced to approximately 30% of that of NT cultures by day 3, and a similar, significant decrease was also observed in the protein expression level of the GluN1 protein on day 3 of culturing, as revealed by PCR and western blotting, respectively (Fig. 7b,d; see also Additional file 1: Fig. S9 in the Online Resource). Transient gene silencing of *GRIN1* also significantly reduced cell proliferation rates (Fig. 7c). The inhibition of chondrogenesis was also demonstrated by a significant decrease in the protein expression of Sox9, and the amount of phosphorylated Sox9 was also reduced (Fig. 7d).

Despite the impaired cartilage ECM production on day 6, a delayed but almost fully recovered cartilage formation occurred by day 10 of culturing in the *GRIN1* siRNA-transfected cultures with 90% efficacy compared to the controls transfected with non-targeting (NT) siRNA (Fig. 7a). Also, the mRNA and protein expression of GluN1 and Sox9, as well as the phosphorylation status of Sox9, have completely recovered by day 10. The proliferation rate in siRNA-treated cultures remained lower along the entire culturing period (Fig. 7b–d).

**Fig. 8:**
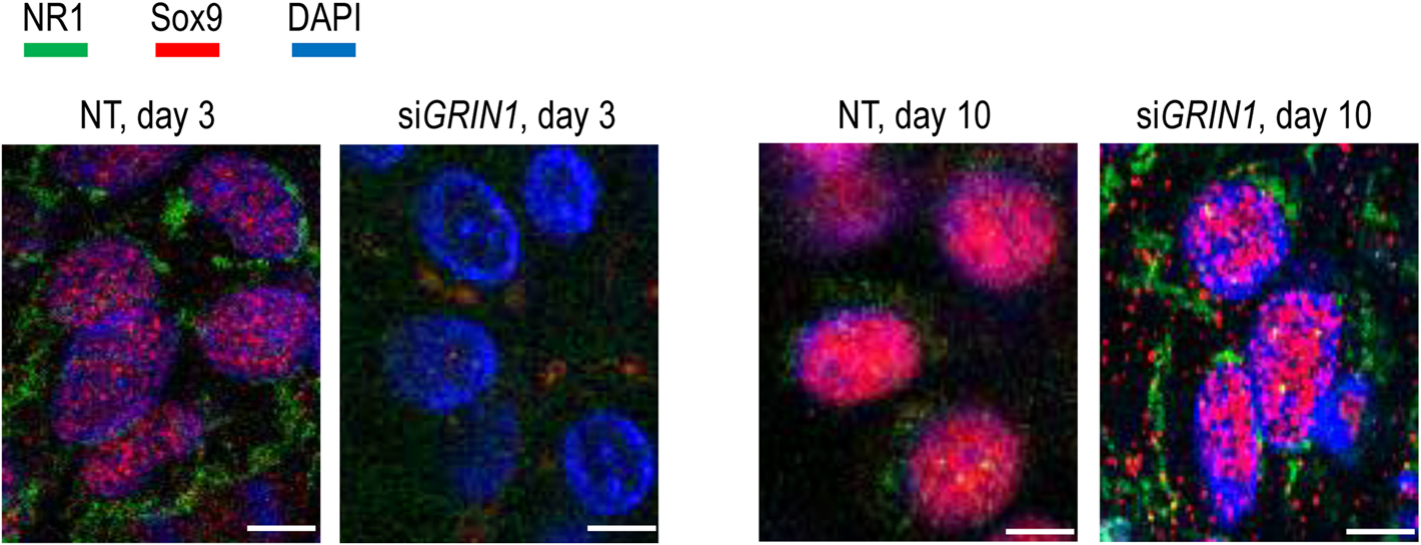
Intracellular localisation of Sox9 and GluN1, following transient gene silencing of *GRIN1* on days 3 and 10. Primary antibodies were visualised with anti-rabbit Alexa Fluor 555 (for Sox9) or anti-mouse Alexa Flour 488 (for GluN1) secondary antibodies. Nuclear DNA was stained with DAPI. Non-targeting (NT) siRNA was used as a control. Images shown are representative out of 3 independent experiments. Scale bar, 15 μm

Transient gene silencing of the GluN1 subunit also interfered with the subcellular localisation of Sox9; on day 3, Sox9 was clearly accumulated in the nucleus of chondrogenic cells in the control cultures, but was almost completely missing from the *GRIN1* knock-down nuclei (Fig. 8). By day 10, however, the nuclear signal of Sox9, along with the signals for the GluN1 subunit, became visible in the differentiated cells of micromass cultures.

## Discussion

Although a growing body of evidence is available on the role of glutamatergic signalling in adult skeletal tissues [20, 27, 28], the involvement of this neurotransmitter and its receptor in the early steps of chondrogenesis leading to the formation of hyaline cartilage has not been investigated. We report for the first time that differentiating chondrogenic cells in micromass cultures express various NMDA-type ionotropic glutamate receptor subunits, both at the mRNA and protein levels. We also found that chicken limb bud-derived chondrogenic cells secrete glutamate into the culture medium during differentiation, and express glutamate transporters VGLUT1 and 2. The detected glutamate concentrations (2–10 nmol/mL) are in a good correlation with what has been reported in cultured rat costal chondrocytes (~ 2–4 nmol/well) [29], and this range is somewhat lower but comparable to those measured in the blood plasma of healthy human individuals [30]. Endogenous glutamate release has also been reported from the human chondrosarcoma cell line SW1353 and from rat articular chondrocytes [21]. Taken together, chondrogenic cells seem to possess a glutamatergic toolkit during the early stages of chondrogenesis.

### NMDAR subunit expression in chondrifying micromass cultures

We established that chicken limb bud-derived chondrogenic cells express GluN1, GluN2B, GluN3A and GluN3B, but not GluN2A subunits, in their plasma membrane, as GluN2A signals could only be detected in the total cell lysates, rather than in the membrane fractions. It is also noteworthy that not all NMDAR subunits showed constant expression profiles. The peak expression of GluN2B coincided with the final commitment and differentiation of chondrogenic cells (i.e., days 2 and 3 of culturing). GluN3A was strongly expressed in more mature cultures, and GluN3B was predominantly present (at least in the membrane fraction) in undifferentiated chondrogenic cells and young chondroblasts. The subunit expression profile detected in chondrifying micromass cultures is in a partial agreement with previously published NMDAR expression data in other models. mRNA expressions for GluN1, GluN2D, and GluN3A subunits were observed in cultured rat costal chondrocytes [22]. Human articular chondrocytes were shown to express GluN1 and GluN2A subunits, whereas OA chondrocytes also possessed GluN2B [19]. In the human chondrocyte cell line SW1353, GluN2D was the most abundant NMDA receptor subunit, although transcripts for GluN1, GluN2A, and GluN2C have also been reported [21]. Osteoblasts, on the other hand, were documented to express GluN1, GluN2D and GluN3B subunits [31]; in contrast, others have described the expression of GluN1 and GluN2C, but not GluN2A, GluN2B or GluN2D [32] or GluN1, GluN2A, GluN2B, and GluN2D, but not GluN2C [15] subunits. Bone marrow-derived MSCs were shown to express all NR subunits, with GluN2C and GluN2D predominating [33].

The above variations in NMDAR subunit expression in both chondrocytes and osteoblasts may be a reflection of differences in the sensitivity or specificity of detection methods used (combinations of conventional and real-time PCR, immunohistochemistry, western blotting and in situ hybridisation), differences in experimental models, or the differentiation status of cells. In particular, Ramage and colleagues reported a switch from GluN2A to GluN2B subunit-containing receptors in human OA chondrocytes, which has been implicated in the disease-associated change in chondrocyte phenotype [19]. It is reasonable to assume that the observed shift in the expression of GluN2B, GluN3A and GluN3B subunits in this study is associated with the differentiation stage of the cells.

### NMDAR-mediated calcium signalling in chondrifying micromass cultures

To measure the ionic flow across the cell membrane following NMDA challenge, whole-cell patch-clamp measurements were performed in voltage-clamp mode. Current traces were recorded while applying a holding potential of − 60 mV. At this membrane potential the possible voltage-gated Na^+^ and K^+^ channels have low opening probabilities, thus the measured current is mainly due to the activity of voltage-independent K^+^ current and the nonspecific leak current [4]. At − 60 mV holding potential the driving force for K^+^ currents is very low, but for the NMDAR-mediated Na^+^ / Ca^2+^ current, it is very high. Application of 300 μM NMDA and 10 μM glycine simultaneously in the extracellular solution, which was previously shown to evoke inward currents in cells transfected with GluN1 and GluN2B subunits [34, 35], had no significant effect on the amplitude of the current. To further test the possible effect of 300 μM NMDA in the presence of 10 μM glycine, voltage ramp protocols were applied where we expected to see the change of reversal potential due to the activation of NMDARs. The agonists had no effect on the reversal potential of the measured current. The lack of observable Ca^2+^ currents could be explained by the miniature nature of the Ca^2+^ signals following NMDA challenge in these cells. Model calculations based on the amplitude of the Ca^2+^ transients (~ 60 nM) and the cell diameter (~ 15–20 μm) predict that for such small signals the Ca^2+^ current through these channels is in the ~ 0.1 pA range, which is well below the resolution limit of whole-cell patch clamp measurements. The calculations are shown in the Online resource (Additional file 1: Supplementary Material).

In order to investigate the functionality of NMDARs live-cell calcium imaging experiments have been performed. In our model, the Ca^2+^ channel(s) activated by NMDA showed only moderate sensitivity and their permeability to Ca^2+^ was fairly low (Ca^2+^ peaks with the amplitude of ~ 60 nM). In contrast, ATP-elicited Ca^2+^-transients in the same model on culturing day 3 were characterised by a relatively high amplitude (~ 180 nM) [36]. This was especially true on day 1 of culturing, when NMDA failed to evoke Ca^2+^ transients. These features are in a good correlation with single channel records obtained in cortical neurons, where the presence of triheterotetrameric NMDARs comprising GluN1, GluN2 and GluN3 subunits was confirmed [37]. Also, the slow inactivation of NMDA-evoked Ca^2+^ signals in micromass cultures was very similar to what has been documented in MSCs following NMDA-challenge [33]. We failed to demonstrate Ca^2+^ signals following application of glycine to chondrogenic cells in our experiments. This suggests that the possibility of the presence of diheterotetrameric channels consisting of GluN1/GluN3A or GluN1/GluN3B subunits is low, since NMDARs with such subunit assembly should confer (a weak) Ca^2+^ permeability to the cells following Gly challenge [11]. Nevertheless, GluN3 subunits offer a membrane potential-independent activation to NMDARs and significantly lower its conductance to Ca^2+^ [10]. These unique features of GluN3 subunit-containing NMDARs are probably very important during the commitment of chondrogenic cells to chondroblasts, when delicate and precisely set changes of intracellular Ca^2+^ concentration are indispensable for chondrogenic differentiation [2].

Chondrogenic cells are known to exhibit spontaneous high-frequency changes of intracellular Ca^2+^ concentration that is sensitive to inhibition of the voltage-gated potassium channel K_V_1.1 [4], and is dependent on functional internal Ca^2+^ stores [5, 38]. We aimed to investigate the possible involvement of NMDARs in the generation of these spontaneous calcium events and found that NMDA has increased the opening probability of Ca^2+^ channels leading to longer and high-amplitude spontaneous events. Moreover, the GluN2B subunit-specific inhibitor ifenprodil almost completely abolished Ca^2+^ oscillations, suggesting that NMDAR-mediated calcium influx was required for the spontaneous Ca^2+^ signalling in chondrogenic cells. The dependence of Ca^2+^ oscillations on NMDARs has already been reported in inferior olivatory neurons of young rats [39], and NMDA has been shown to increase the amplitude of oscillatory Ca^2+^ events in developing hippocampal cells [40]. In contrast, NMDARs provided only a minor contribution to Ca^2+^ oscillations in cultured neocortical neurons [41]. Nonetheless, this is the first study to implicate the role of NMDARs in Ca^2+^ oscillations of a differentiating non-neural cell type.

### Pharmacological modulation of NMDAR activity influences chondrogenesis

According to our previous results, in vitro chondrogenesis in micromass cultures is sensitive to manipulation of the precisely set temporal pattern observed in the intracellular Ca^2+^ concentration [2]. We therefore wanted to examine the possible effects of NMDAR agonists and inhibitors on chondrogenesis. When NMDA (either alone or in combination with glycine) was present throughout the entire culturing period, the amount of metachromatic cartilage matrix remained unchanged. In contrast, treatments with glycine alone stimulated cartilage ECM formation even when GlyRs were concurrently blocked with strychnine. When applied alone, strychnine had a significant inhibitory effect on cartilage formation, reflecting on the importance of ionic flux via strychnine-sensitive GlyRs in chondrogenesis. GlyRs are chloride channels, which cause hyperpolarization in neurons [42]. Chloride channels have been previously reported in chondrocytes [7], and we have identified a wide array of GlyR transcripts in chondrifying cultures on various days of culturing (see Fig. S4 in the Online Resource). The observed compensatory effect of glycine on cultures concurrently blocked with strychnine was possibly mediated through Gly-sensitive NMDARs and/or other mechanisms. The antiproliferative effect of glycine, despite significantly enhanced chondrogenic differentiation, remains unexplored; a possible mechanism involves Cl^−^ influx, leading to hyperpolarisation and subsequent modulation of cell proliferation [43, 44]. The fact that we did not observe similar effects to the results of the proliferation assay with the MTT assay is attributable to the fact that the latter was used as a short-term test to assay the immediate effects of the compounds on mitochondrial activity (a measure of cell viability).

Although administration of NMDA failed to modulate cartilage matrix formation, the GluN2 subunit blocker ifenprodil caused a pronounced inhibition of chondrogenesis, suggesting that a baseline Ca^2+^ flux through GluN2B subunit-containing receptors was crucial for the progression of chondrogenesis. In addition, ifenprodil significantly reduced proliferation rate, which could have further exacerbated its anti-chondrogenic effects. When DCKA, the competitive antagonist of the Gly-site on the GluN1 subunit was applied, a massive elevation of cartilage ECM production was detected, in addition to the activation of the chondrogenic transcription factor Sox9. This could have been caused by NMDAR-unrelated effects of DCKA. Kynurenic acid is a tryptophan metabolite, which is now accepted to act on an array of cellular targets. For example, it is a potent inhibitor of cytosolic sulfotransferases (SULTs) involved in the metabolism of various substances including certain hormones [45], and it also inhibits the poly(ADP)ribose-polymerase PARP enzyme [46]. PARP has been shown to play an important regulatory role in the chondrogenic differentiation of micromass cultures; the PARP-inhibitor 3-AB caused a similarly enhanced cartilage ECM production [47]. Also, given that DCKA and ifenprodil have different targets (i.e. the former could have inhibited all NMDARs containing the GluN1 subunit, the latter probably only inhibited the GluN2B-containing receptors), the divergent effects of the two inhibitors observed in the present study support studies in the OA field indicating that GluN2B and GluN2A-containing subunits (and the ionic fluxes mediated by these channels) have different effects [19]. Nevertheless, the specific mechanism of the massively enhanced chondrogenic differentiation caused by DCKA remains elusive.

### Transient gene silencing of GRIN1 temporarily halts chondrogenesis

As the application of ifenprodil and DCKA resulted in opposing effects on chondrogenesis, we undertook to transiently knock down the GluN1 subunit using siRNA. Given that functional NMDARs require GluN1 subunit for assembly and membrane trafficking [48], the transient knock down of GluN1 subunits essentially abolished (i.e. reduced to less than 30% of that found in cultures transfected with the non-targeting siRNA construct) functional NMDARs in chondrogenic cells, compared to the non-targeting control. In these cultures, cartilage formation was almost completely blocked, along with a significantly reduced cell proliferation, but no alterations in cellular viability was detected. Essentially, the knock-down experiments substantiated parallel experiments in which ifenprodil produced similar effects. The antiproliferative effect of *GRIN1* gene silencing is consistent with that has been observed in SW1353 cells and rat articular chondrocytes following *GRIN1* gene silencing [21]. This suggests that chondrogenic cells lacking functional NMDARs, although still viable, are unable to differentiate into ECM-producing chondroblasts. A possible explanation lies in the dependence of chondrogenic differentiation on a significant but transient rise in cytosolic Ca^2+^ levels [25]; suggesting that Ca^2+^ flux via functional NMDARs was essential for the above-described mechanism.

Indeed; along with the re-expression of the GluN1 protein after the transient effects of the siRNA construct diminished, micromass cultures gained back their ability to produce cartilage ECM, and complete their differentiation programme by day 10 of culturing. The significantly downregulated NMDAR expression and function kept chondroprogenitors in an early, undifferentiated stage and delayed the nuclear accumulation of Sox9 for a few days. When, however, the effects of transient gene silencing have diminished, the chondrogenic differentiation programme has commenced, along with the nuclear accumulation of Sox9.

These data are in agreement with the fact that GluN1-null mice were described as non-viable and died before birth [26]. In costal chondrocytes isolated from early GluN1-null foetuses various defects were detected including a significantly decreased alkaline phosphatase (ALP) activity, also suggesting a key role of functional NMDARs in the regulation of skeletogenesis [26].

## Conclusions

In conclusion, this is the first study to show that NMDA-mediated signalling may act as a pivotal factor in embryonic limb bud-derived chondrogenic cultures. We have shown that function of NMDARs consisting of GluN1/GluN2B/GluN3 subunits are required for chondrogenesis, and play an essential role in Ca^2+^ oscillations of differentiating chondrogenic cells. Moreover, transient knock-down of GluN1 mRNA expression, as well as pharmacological inhibition of NMDAR function temporarily blocked the differentiation of chondroprogenitor cells. Our findings complement and extend the results of other groups, who demonstrated NMDAR activity in adult articular chondrocytes with possible roles in mechanotransduction, and have also shown that NMDARs are characterised by altered composition and function during inflammatory joint diseases such as osteoarthritis.

## Supplementary information

**Additional file 1: Table S1.** Sequences of primer pairs and PCR condition. **Table S2.** List of transcript variants amplified by the *GRIN* primers. **Figure S1.** Specificity controls for anti-GluN antibodies employed in this study. **Figure S2.** Uncropped agarose gel images showing negative results for *GRIN2C* and *GRIN2D* (RT-PCR) and lack of GluN2A signals in the membrane fraction (western blot). **Figure S3.** Visualising GluN subunit expression using immunocytochemistry. **Figure S4.** AMPA, kainate, and metabotropic GluR, as well as GlyR subunit mRNA expression profiles in chondrogenic micromass cultures. **Figure S5.** Glutamate secretion into the medium during chondrogenesis. **Figure S6.** VGLUT1 and VGLUT2 expression profiles during chondrogenesis. **Figure S7.** Negative control experiments for Fura-2 based fluorescence calcium assays . **Figure S8.** Whole-cell patch-clamp measurements in chondrogenic cells. **Figure S9.** mRNA expression profiles of chondrogenic marker genes following gene silencing by *GRIN1* siRNA.

## Data Availability

Data sharing is not applicable to this article as no datasets were generated or analysed during the current study. All other data generated or analysed during this study are included in this published article [and its Additional file 1: supplementary information files].

## Abbreviations

BM-MSC: Marrow-derived mesenchymal stem cell
DCKA: 5,7-dichlorokynurenic acid
DMMB: Dimethyl-methylene blue
ECM: Extracellular matrix
FTHM: Full time at half maximum
GlyR: Glycine receptor
NMDAR: *N*-methyl-D-aspartate receptor
SOCE: Store-operated calcium entry
TB: Toluidine blue
VGLUT: Vesicular glutamate transporter
