# Supplementary material for: N-methyl-D-aspartate (NMDA) receptor expression and function is required for early chondrogenesis
Source: Cell Commun Signal. 2019 Dec 16;17:166. doi: 10.1186/s12964-019-0487-3 (PMC6915923; doi:10.1186/s12964-019-0487-3)
Supplement: Supplementary file 1 — Additional file 1: Table S1. Sequences of primer pairs and PCR condition. Table S2. List of transcript variants amplified by the GRIN primers. Figure S1. Specificity controls for anti-GluN antibodies employed in this study. Figure S2. Uncropped agarose gel images showing negative results for GRIN2C and GRIN2D (RT-PCR) and lack of GluN2A signals in the membrane fraction (western blot). Figure S3. Visualising GluN subunit expression using immunocytochemistry. Figure S4. AMPA, kainate, and metabotropic GluR, as well as GlyR subunit mRNA expression profiles in chondrogenic micromass cultures. Figure S5. Glutamate secretion into the medium during chondrogenesis. Figure S6. VGLUT1 and VGLUT2 expression profiles during chondrogenesis. Figure S7. Negative control experiments for Fura-2 based fluorescence calcium assays . Figure S8. Whole-cell patch-clamp measurements in chondrogenic cells. Figure S9. mRNA expression profiles of chondrogenic marker genes following gene silencing by GRIN1 siRNA. [file 12964_2019_487_MOESM1_ESM.docx]

Supplementary Materials for

***N*-methyl-D-aspartate (NMDA) receptor expression
and function is required for early chondrogenesis**

Csaba Matta^#^, Tamás Juhász, János Fodor, Tibor Hajdú, Éva Katona,
Csilla Szűcs-Somogyi, Roland Takács, Judit Vágó, Tamás Oláh, Ádám Bartók, Zoltán Varga, György Panyi, László Csernoch, Róza Zákány^#^

^#^Corresponding authors. E-mail: matta.csaba@med.unideb.hu (C.M.), roza@anat.med.unideb.hu (R.Z.)

**This PDF file includes:**

- **Table S1.** Sequences of primer pairs and PCR conditions
- **Table S2.** List of transcript variants amplified by the *GRIN* primers
- **Figure S1.** Specificity controls for anti-GluN antibodies employed in this study
- **Figure S2.** Uncropped agarose gel images showing negative results for *GRIN2C* and
   *GRIN2D* (RT-PCR) and lack of GluN2A signals in the membrane fraction
   (western blot)
- **Figure S3.** Visualising GluN subunit expression using immunocytochemistry
- **Figure S4.** AMPA, kainate, and metabotropic GluR, as well as GlyR subunit mRNA
   expression profiles in chondrogenic micromass cultures
- **Figure S5.** Glutamate secretion into the medium during chondrogenesis
- **Figure S6.** VGLUT1 and VGLUT2 expression profiles during chondrogenesis
- **Figure S7.** Negative control experiments for Fura-2 based fluorescence calcium assays
- **Figure S8.** Whole-cell patch-clamp measurements in chondrogenic cells
- **Figure S9.** mRNA expression profiles of chondrogenic marker genes following
   gene silencing by *GRIN1* siRNA
- Calculations for estimating the Ca^2+^ current amplitude based on the parameters of Ca^2+^ transients obtained during fluorescent single cell Ca^2+^ imaging

*1. Sequences of primer pairs for PCRs used in the study*

**Table S1.** Nucleotide sequences, amplification sites, GenBank accession numbers, amplimer sizes and PCR reaction conditions for each primer pair are shown.

| ***Gene*** | *Primer* | *Nucleotide sequence (5’→3’)* | *GenBank  Accession No.* | *Annealing temperature* | *Amplimer size (bp)* |
| --- | --- | --- | --- | --- | --- |
| ***ACAN*** | sense | CAATGCAGAGTACAGAGA (1767–1784) | **XM_015291979.2** | 54 °C | 429 |
|  | antisense | TCTGTCTCACGGACACCG (2178–2195) |  |  |  |
| ***GRIA1*** | sense | TCAGACGAATGAGTTTGGGATA (1789–1810) | **NM_001001774** | 54 °C | 174 |
|  | antisense | AGGAAGGCAGCCAGGTTA (1945–1962) |  |  |  |
| ***GRIA2*** | sense | TCTGGGATTTACTGATGG (932–949) | **NM_001001775** | 47 °C | 326 |
|  | antisense | ATGCCCTTTCTATTTCTAC (1239–1257) |  |  |  |
| ***GRIA3*** | sense | GCAGAGGCTTTCCGTTAC (1048–1065) | **NM_001112799** | 47 °C | 272 |
|  | antisense | GTTGGCACAAATCTTTCATA (1300–1319) |  |  |  |
| ***GRIA4*** | sense | CCCTTTGGCGTATGAGAT (1635–1652) | **NM_001113186** | 52 °C | 265 |
|  | antisense | GAACCACCAGACACCTCC (1882–1899) |  |  |  |
| ***COL2A1*** | sense | GGACCCAAAGGACAGACGG (1191–1209) | [**NM_204426**](http://www.ncbi.nlm.nih.gov/entrez/viewer.fcgi?db=nucleotide&val=45383308) | 59 °C | 401 |
|  | antisense | TCGCCAGGAGCACCAGTT (1574–1591) |  |  |  |
| ***GAPDH*** | sense | GAGAACGGGAAACTTGTCAT (238–257) | **NM_204305** | 54 °C | 556 |
|  | antisense | GGCAGGTCAGGTCAACAA (776–793) |  |  |  |
| ***GLRA1*** | sense | CCTCCTCATTGTCATTCTGT (6220–6239) | **XM_025155016.1** | 52 °C | 276 |
|  | antisense | TGCCTCCTCTTCCTTCTG (6478–6495) |  |  |  |
| ***GLRA2*** | sense | TTTGGCTACACTATGAATG (1193–1211) | **XM_001234290** | 48 °C | 253 |
|  | antisense | AGAAAGAAACCCAGGACA (1428–1445) |  |  |  |
| ***GLRA3*** | sense | AACATCTTTCTCCGTCAG (328–345) | **XM_420527** | 50 °C | 357 |
|  | antisense | TGCTACTTGAACTGCTCC (667–694) |  |  |  |
| ***GLRA4*** | sense | ATCACCACCGTGCTCACC (1031–1048) | **XM_015278390.2** | 56 °C | 258 |
|  | antisense | CCCGTAGCCTCGGAAGTAG (1270–1288) |  |  |  |
| ***GLRB*** | sense | GGGTGTTTATGCTCCTAC (1008–1025) | **XM_420379** | 50 °C | 354 |
|  | antisense | GTGCCATTCACAGTGTTC (1344–1361) |  |  |  |
| ***GRIN1*** | sense | AGGAGAGAGAGTCCAAGGCT (560–579) | **NM_206979.1** | 56 °C | 307 |
|  | antisense | ACCGCCACTGCATCACTTAT (847–866) |  |  |  |
| ***GRIN2A*** | sense | GCCACATGATTATTCACCTC (2817–2836) | **XM_025155260.1** | 49 °C | 373 |
|  | antisense | CAGCCACAGGGTTTCTAACT (3170–3189) |  |  |  |
| ***GRIN2B*** | sense | AAAAGTTCCAGAAACCCAA (2791–2809) | **XM_015289359.2** | 50 °C | 402 |
|  | antisense | TGTGACAAATGCCAGTGAG (3174–3192) |  |  |  |
| ***GRIN2C*** | sense | ACCCTGACCCATTTTTCCCG (5068–5087) | **XM_025141838.1** | 50 °C | 303 |
|  | antisense | GATTCCGGTCTCCACGAAGG (5351–5370) |  |  |  |
| ***GRIN2D*** | sense | GCAGCAATTACCCGGCCA (2090–2107) | **XM_014262098*** | 52 °C | 393 |
|  | antisense | CCAGCAGCATGTAGAACACCC (2462–2482) |  |  |  |
| ***GRIN3A*** | sense | AGCCTGAACTTGCTCTTATT (1271–1290) | **XM_001232181** | 52.5 °C | 484 |
|  | antisense | ACGGTGTCATTTCCTCCT (1737–1754) |  |  |  |
| ***GRIN3B*** | sense | GGTGGAGCATCCCTTCGT (1309–1326 | **XM_015299990.2** | 57 °C | 177 |
|  | antisense | TACCCATAGCAGCACTTCTTGT (1464–1485) |  |  |  |
| ***GRM1*** | sense | ACATCCCACAAATTGCCTAC (784–803) | **XM_015284288.2** | 54 °C | 350 |
|  | antisense | CGTCATGCCCTCACAGAA (1116–1133) |  |  |  |
| ***GRM2*** | sense | GACTTCTACCAAGCCAAAGC (808–827) | **XM_015292870.2** | 55 °C | 246 |
|  | antisense | GAAGAGCACGACCACCCT (1036–1053) |  |  |  |
| ***GRM3*** | sense | TGTCTGCTATTCTGCCCTCC (2336–2355) | **XM_015299810.2** | 57 °C | 485 |
|  | antisense | GCACCACAAAGCCGCTCA (2803–2820) |  |  |  |
| ***GRM4*** | sense | CCGGAGAAGGGGTCGGA (156–172) | [**XM_015298989.2**](https://www.ncbi.nlm.nih.gov/entrez/viewer.fcgi?db=nucleotide&id=1390094894) | 57 °C | 311 |
|  | antisense | GCAGAAAGAGAGACGCTCCA (447–466) |  |  |  |
| ***GRM5*** | sense | AGGCTGTCGGTGGAATAA (977–995) | **NM_204138** | 55 °C | 214 |
|  | antisense | TGCTGCGTCCTCAGTGTC (1172–1190) |  |  |  |

**Table S1.** *(continued)*

| ***Gene*** | *Primer* | *Nucleotide sequence (5’→3’)* | *GenBank  Accession No.* | *Annealing temperature* | *Amplimer size (bp)* |
| --- | --- | --- | --- | --- | --- |
| ***GRM6*** | sense | ACGCTAACCGTGTCCTTGAG (2516–2535) | **NM_000843.4^#^** | 58 °C | 379 |
|  | antisense | GCATGAAGCTCACATGCTGG (2875–2894) |  |  |  |
| ***GRM7*** | sense | AGGGATTTGATGCCTACTTC (1129–1148) | **XM_004944718.3** | 54 °C | 436 |
|  | antisense | GTGCTGGTGTTGGTGGTG (1547–1564) |  |  |  |
| ***GRM8*** | sense | TAACACCAGGTATGACTTCT (1177–1196) | **XM_425426** | 49 °C | 379 |
|  | antisense | CCAACTATCTGAGCCAAT (1538–1555) |  |  |  |
| ***GRIK1*** | sense | GATGTACGACGCAGTTTA (1053–1070) | **XM_025144972.1** | 50 °C | 364 |
|  | antisense | CAGCGAGTCTGTGATGTT (1399–1416) |  |  |  |
| ***GRIK2*** | sense | TTCCCTGTCCAATCGTTC (1835–1852) | **XM_025149033.1** | 54 °C | 215 |
|  | antisense | ACTGTCCACTGGCATCCT (2032–2049) |  |  |  |
| ***GRIK3*** | sense | CCAGTGGAACGGGATGAT (1494–1511) | **XM_004947758.3** | 54 °C | 229 |
|  | antisense | CAGGTAGGCGAGGAGAATGT (1703–1722) |  |  |  |
| ***GRIK4*** | sense | GCTCGCTTGACGCCCTAT (1817–1834) | **XM_015298177.2** | 56 °C | 129 |
|  | antisense | GCCTTGTTGCATGAACCC (1928–1945) |  |  |  |
| ***GRIK5*** | sense | GTCAGCACTGTGATGCGG (562–579) | **XM_017740731.1^¶^** | 56 °C | 201 |
|  | antisense | GTCAGCTTTCCGGTTGATGAG (742–762) |  |  |  |
| ***SOX9*** | sense | CCCCAACGCCATCTTCAA (713–731) | [**NM_204281**](http://www.ncbi.nlm.nih.gov/entrez/viewer.fcgi?db=nucleotide&val=45383571) | 54 °C | 381 |
|  | antisense | CTGCTGATGCCGTAGGTA (1076–1093) |  |  |  |
| ***SLC17A7*** | sense | TGGAATGTGCTGCAAACAACC (1692–1712) | **XM_015616358.1*** | 54 °C | 168 |
|  | antisense | CGGCCGCTCCTTGTATTTAT (1840–1859) |  |  |  |
| ***SLC17A6*** | sense | TCGGGATCCGATGTAACTTGG (459–479) | **NM_001168383.1** | 50 °C | 106 |
|  | antisense | TTCGCTTTAAGGGTTCCCACC (544–564) |  |  |  |

*The chicken (*Gallus gallus*) sequence was not available for this transcript in GenBank at the time of primer design
(March 2019), so the primers have been designed to amplify the same sequence in the available closest relative (*Pseudopodoces humilis*).

^#^The chicken (*Gallus gallus*) sequence was not available for this transcript in GenBank at the time of primer design
(March 2019), and the sequence was not available in the closest relatives (*Aves*) either, so the primers have been designed to amplify the human sequence (*Homo sapiens sapiens*).

^¶^The chicken (*Gallus gallus*) sequence was not available for this transcript in GenBank at the time of primer design
(March 2019), so the primers have been designed to amplify the same sequence in the available closest relative
(*Corvus brachyrhynchos*).

*2. List of transcript variants amplified by the GRIN primers*

**Transcript variants for the GRIN1 primer pair:**

>[XM_015279349.2](https://www.ncbi.nlm.nih.gov/entrez/viewer.fcgi?db=nucleotide&id=1390077582) PREDICTED: Gallus gallus glutamate ionotropic receptor NMDA type subunit 1 (GRIN1), transcript variant X2, mRNA

product length = 307

Forward primer 1 AGGAGAGAGAGTCCAAGGCT 20

Template 554 .................... 573

Reverse primer 1 ACCGCCACTGCATCACTTAT 20

Template 860 .................... 841

>[XM_015279362.1](https://www.ncbi.nlm.nih.gov/entrez/viewer.fcgi?db=nucleotide&id=971422734) PREDICTED: Gallus gallus glutamate ionotropic receptor NMDA type subunit 1 (GRIN1), transcript variant X12, mRNA

product length = 307

Forward primer 1 AGGAGAGAGAGTCCAAGGCT 20

Template 554 .................... 573

Reverse primer 1 ACCGCCACTGCATCACTTAT 20

Template 860 .................... 841

>[XM_015279356.1](https://www.ncbi.nlm.nih.gov/entrez/viewer.fcgi?db=nucleotide&id=971422724) PREDICTED: Gallus gallus glutamate ionotropic receptor NMDA type subunit 1 (GRIN1), transcript variant X8, mRNA

product length = 307

Forward primer 1 AGGAGAGAGAGTCCAAGGCT 20

Template 554 .................... 573

Reverse primer 1 ACCGCCACTGCATCACTTAT 20

Template 860 .................... 841

**Transcript variants for the GRIN2A primer pair:**

>[XM_025155259.1](https://www.ncbi.nlm.nih.gov/entrez/viewer.fcgi?db=nucleotide&id=1390074330) PREDICTED: Gallus gallus glutamate ionotropic receptor NMDA type subunit 2A (GRIN2A), transcript variant X3, mRNA

product length = 373

Forward primer 1 GCCACATGATTATTCACCTC 20

Template 2817 .................... 2836

Reverse primer 1 CAGCCACAGGGTTTCTAACT 20

Template 3189 .................... 3170

>[XM_015294579.2](https://www.ncbi.nlm.nih.gov/entrez/viewer.fcgi?db=nucleotide&id=1390074329) PREDICTED: Gallus gallus glutamate ionotropic receptor NMDA type subunit 2A (GRIN2A), transcript variant X2, mRNA

product length = 373

Forward primer 1 GCCACATGATTATTCACCTC 20

Template 3034 .................... 3053

Reverse primer 1 CAGCCACAGGGTTTCTAACT 20

Template 3406 .................... 3387

>[XM_025155258.1](https://www.ncbi.nlm.nih.gov/entrez/viewer.fcgi?db=nucleotide&id=1390074327) PREDICTED: Gallus gallus glutamate ionotropic receptor NMDA type subunit 2A (GRIN2A), transcript variant X1, mRNA

product length = 373

Forward primer 1 GCCACATGATTATTCACCTC 20

Template 2817 .................... 2836

Reverse primer 1 CAGCCACAGGGTTTCTAACT 20

Template 3189 .................... 3170

**Transcript variants for the GRIN2B primer pair:**

[There are no transcript variants.]

**Transcript variants for the GRIN2C primer pair:**

>[XM_015295464.2](https://www.ncbi.nlm.nih.gov/entrez/viewer.fcgi?db=nucleotide&id=1390080123) PREDICTED: Gallus gallus glutamate ionotropic receptor NMDA type subunit 2C (GRIN2C), transcript variant X3, mRNA

product length = 303

Forward primer 1 ACCCTGACCCATTTTTCCCG 20

Template 2016 .................... 2035

Reverse primer 1 GATTCCGGTCTCCACGAAGG 20

Template 2318 .................... 2299

>[XM_025141839.1](https://www.ncbi.nlm.nih.gov/entrez/viewer.fcgi?db=nucleotide&id=1390080121) PREDICTED: Gallus gallus glutamate ionotropic receptor NMDA type subunit 2C (GRIN2C), transcript variant X2, mRNA

product length = 303

Forward primer 1 ACCCTGACCCATTTTTCCCG 20

Template 2077 .................... 2096

Reverse primer 1 GATTCCGGTCTCCACGAAGG 20

Template 2379 .................... 2360

**Transcript variants for the GRIN2D primer pair:**

[There are no transcript variants.]

**Transcript variants for the GRIN3A primer pair:**

[There are no transcript variants.]

**Transcript variants for the GRIN3B primer pair:**

>[XM_015299989.2](https://www.ncbi.nlm.nih.gov/entrez/viewer.fcgi?db=nucleotide&id=1390097055) PREDICTED: Gallus gallus glutamate ionotropic receptor NMDA type subunit 3B (GRIN3B), transcript variant X2, mRNA

product length = 177

Forward primer 1 GGTGGAGCATCCCTTCGT 18

Template 1309 .................. 1326

Reverse primer 1 TACCCATAGCAGCACTTCTTGT 22

Template 1485 ...................... 1464

>[XM_015299988.2](https://www.ncbi.nlm.nih.gov/entrez/viewer.fcgi?db=nucleotide&id=1390097053) PREDICTED: Gallus gallus glutamate ionotropic receptor NMDA type subunit 3B (GRIN3B), transcript variant X1, mRNA

product length = 177

Forward primer 1 GGTGGAGCATCCCTTCGT 18

Template 1306 .................. 1323

Reverse primer 1 TACCCATAGCAGCACTTCTTGT 22

Template 1482 ...................... 1461

*3. Specificity controls for anti-GluN antibodies employed in this study*

To check the specificity of the antibodies used for the detection of GluN subunits in chicken HD cultures, we also prepared a lysate from chicken brain and probed both samples with the antibodies. The results of the specificity control experiments are shown below.


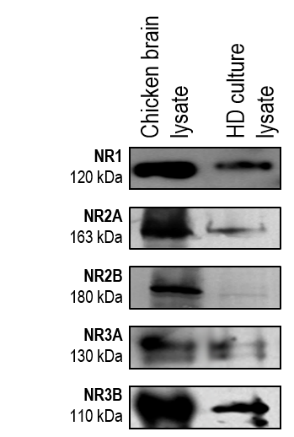


**Figure S1.** Chicken brain lysates were run alongside lysates prepared from HD cultures (both set to identical protein concentrations at 1 μg/mL) and probed with the anti-GluN antibodies. Gels were blotted and membranes were developed as described in the manuscript.

*4. Uncropped gel images showing negative results for GRIN2C* and *GRIN2D (RT-PCR) and lack of GluN2A signals in the membrane fraction (western blot)*

In Figure 1 of the manuscript, only those NMDAR subunits are presented whose expression was confirmed at the transcript level (by RT-PCR). Figure S2A shows the negative results for the analysis of genes encoding GluN2C and GluN2D subunits (*GRIN2C* and *GRIN2D*). In Figure 2B, we found no specific bands at the expected MW (160 kDa) ­– a representative membrane image is shown in Figure S2B.


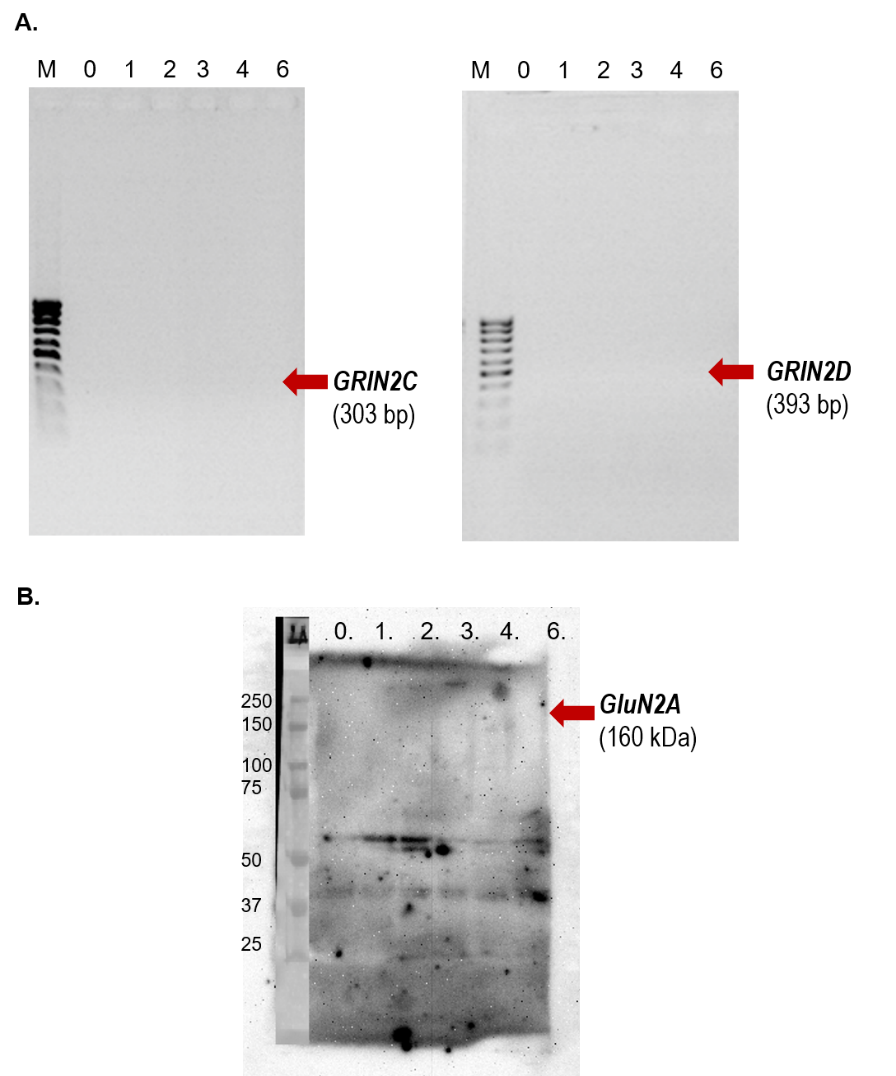


**Figure S2.** Lack of signals in agarose gels for *GRIN2C* and *GRIN2D* following amplification (**A**), and lack of signals in the membrane fraction after probing with the anti-GluN2A antibody (**B**).

*5. Visualising GluN subunit expression using immunocytochemistry*

To further confirm the presence of GluN1, GluN2B, GluN3A and GluN3B NMDAR subunits in differentiating cells of HDC, immunocytochemistry was performed on 3-day-old cultures. After fixing the cultures in 4% paraformaldehyde and blocking the nonspecific binding sites with 1% BSA, cultures were incubated with the first primary antibody (anti-GluN1 or anti-GluN2B, both polyclonal produced in rabbit) at 4^o^C overnight. Then, biotinylated goat anti-rabbit antibody (Vector Laboratories, Burlingame, CA, USA) was added to the cultures, at a dilution of 1:1000, at room temperature for 2 h. Then, the second primary antibody (anti-GluN2B, anti-GluN3A or anti-GluN3B, all polyclonal produced in rabbit) was added an incubated at 4^o^C overnight. On the third day, the biotinylated goat antibody was visualized with Streptavidin Alexa Fluor 488 conjugate, while the second set of primary antibodies were visualized with anti-rabbit Alexa Fluor 555 secondary antibody (Life Technologies Corporation, Carlsbad, CA, USA) at a dilution of 1:1000. Cultures were mounted in Vectashield mounting medium (Vector Laboratories) containing DAPI for nuclear DNA staining. Photomicrographs of the cultures were taken with an Olympus FV3000 confocal microscope (Olympus Co., Tokyo, Japan) using a 60x PlanApo N oil-immersion objective (NA: 1.42) and FV31S-SW software (Olympus Co., Tokyo, Japan).

| **Antibody** | **Dilution** | **Vendor, Cat. No.** |
| --- | --- | --- |
| Anti-GluN1 | 1:50 | Cell Signaling, #5704S |
| Anti-GluN2B | 1:50 | Cell Signaling, #4207S |
| Anti-GluN3A | 1:50 | Alomone Labs, AGC-030 |
| Anti-GluN3B | 1:50 | Alomone Labs, AGC-031 |

Whilst these experiments confirmed the presence and co-localisation of the GluN3A and GluN3B subunits with both GluN1 and GluN2B subunits (Figure S3), they do not allow distinguishing between triheterotetrameric and diheterotetrameric NMDAR compositions.


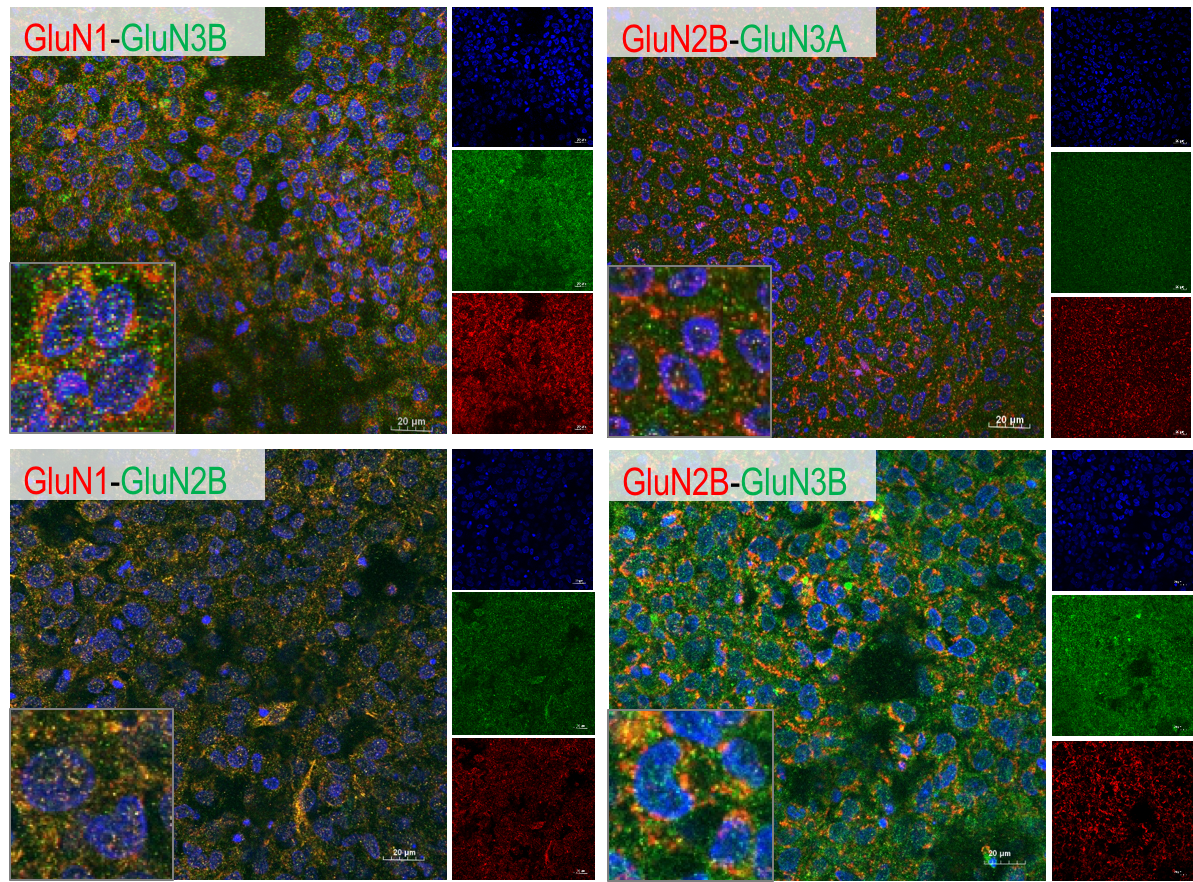


**Figure S3.** Intracellular localisation and distribution of GluN1, GluN2B, GluN3A, and GluN3B subunits in 3-day-old cultures. The first set of primary antibodies were visualised with Streptavidin Alexa Fluor 488 conjugate (green), while the second set of primary antibodies were visualized with anti-rabbit Alexa Fluor 555 secondary antibody (red). Nuclear DNA was stained with DAPI. Scale bar, 20 µm

*6. Chondrogenic cells express various iGluRs (AMPA, kainate), mGluRs and GlyRs at the mRNA level*

In addition to NMDARs, glutamate and glycine can also act on other types of receptors. Therefore, conventional RT-PCR reactions were performed to detect mRNA expression patterns of other ionotropic glutamate receptors (AMPA and kainate), metabotropic glutamate receptors (mGluR1–8) and glycine receptors (GlyRA1–4 and GlyRB). Primer pairs used to amplify specific sequences are shown in *Table S1*. Note that sequence information for certain genes (*GRIN2D, GRM6, GRIK5* and *SLC17A7* [VGLUT1]) were not available in GenBank at the time of primer design (March 2019), the primers have been designed on the intended sequence in the closest available relative species.

mRNAs of the majority of these receptors were detectable at least on certain days of culturing, while some others were present throughout the 6-day-long culturing (*Fig. S4*). As we aimed at investigating the function of NMDA receptors only during *in vitro* chondrogenesis in this work, protein expression analyses of the above-mentioned receptors were not performed. Owing to the diverse expression profile of various glutamate receptors in micromass cultures, we choose not to apply glutamate as an agonist during our experiments; moreover, the effects of glycine acting on NMDARs were investigated with simultaneous inhibition of GlyRs by strychnine.


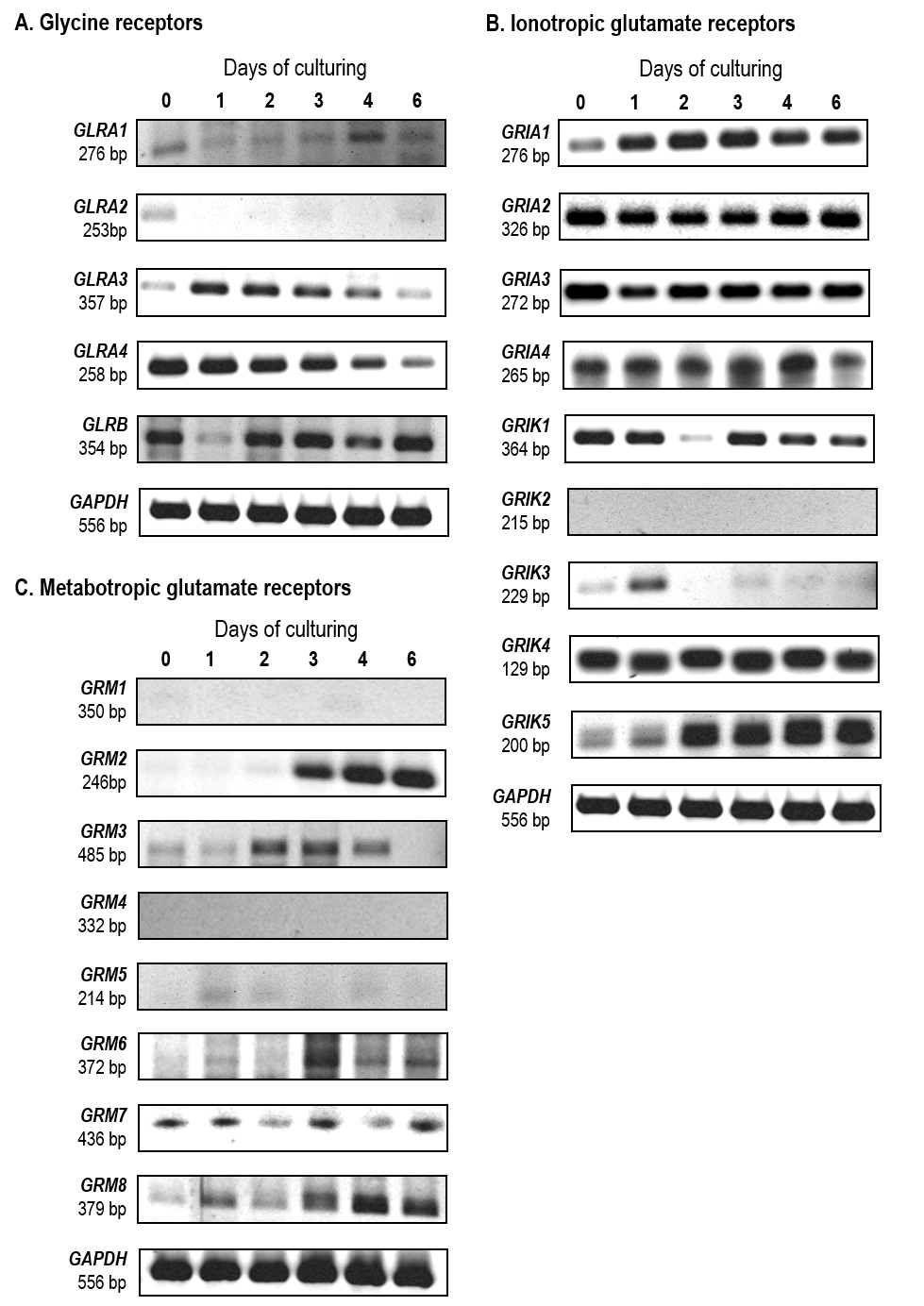


**Figure S4.** Glycine receptor (GlyR) (**A**), ionotropic glutamate receptor (iGluR; AMPA and kainate receptors) (**B**), and metabotropic glutamate receptor (mGluR) (**C**) subunit mRNA expression profiles in chondrifying chicken micromass cultures during the entire culturing period (days 0–6) (*n*=3 for each culturing day).

*7. Glutamate release by chondrifying cells into the culture medium*

**
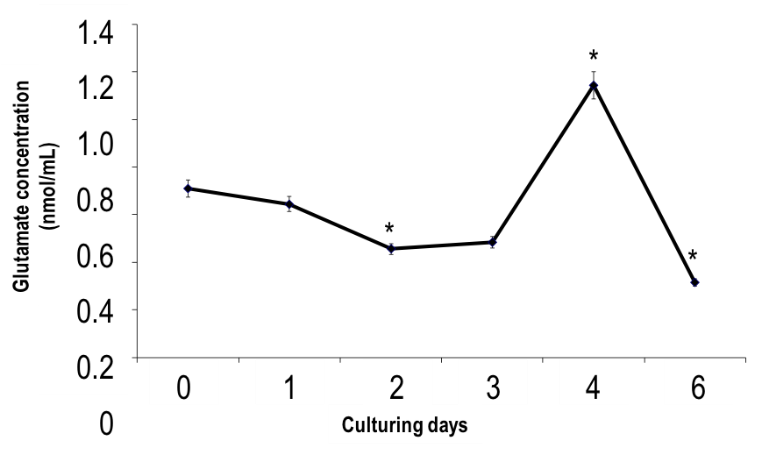
**

**Figure S5.** Glutamate release by chondrifying micromass cultures during chondrogenesis into the culture medium as determined by a glutamine/glutamate colorimetric assay. Statistically significant (*P*<0.05) differences in the amount of released glutamate compared to the previous day are marked by asterisks (*). Measurements were performed on 4 parallel samples on each culturing day in 3 independent experiments; representative data are shown.

*8. Expression of vesicular glutamate transporters (VGLUT) during chondrogenesis*


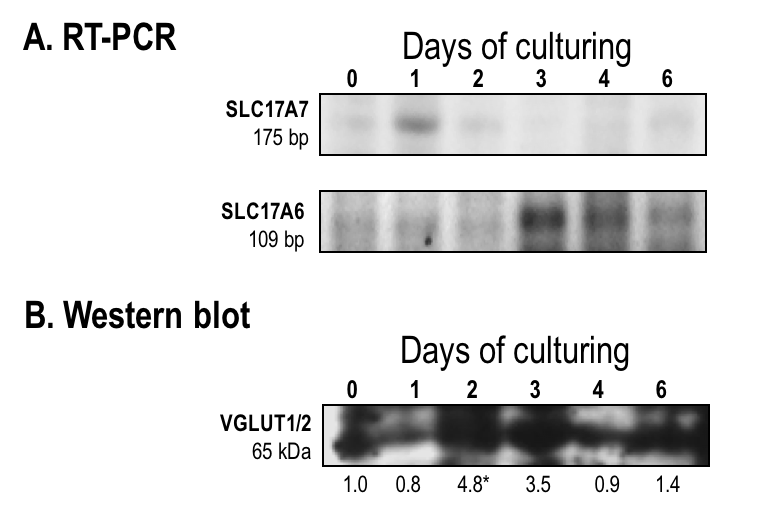


**Figure S6.** VGLUT expression profile of chondrifying chicken micromass cultures during the entire culturing period (days 0–6). **A.** mRNA expression profile of SLC17A7 (VGLUT1) and SLC17A6 (VGLUT2). **B.** Protein expression profile of VGLUT1/2 in total cell lysates. Representative images out of 3 independent experiments, each showing a similar expression profile. Significant (<0.05) alteration in protein levels of the mean signal densities from 3 experiments relative to day 0 (mean) is marked by asterisk (*).

*9. Negative control experiments for Fura-2 based fluorescence calcium assays*

The ratiometric dye Fura-2 was used to detect the changes in intracellular calcium concentration. Given that following local administration of NMDA constant increase in fluorescence intensity was detected, in order to exclude artefacts, we performed two additional experiments. (1) In both 2- and 3-day-old cultures, the fluorescence ratio (following excitation at 340/380 nm) was stable over time in our measurements. (2) To exclude that the rise in fluorescence intensity was not attributable to mechanical forces caused by the local application of solvents, we present negative control recordings, where Tyrode’s solution was applied locally to the cell, causing no aspecific elevations in cytosolic Ca^2+^ levels.


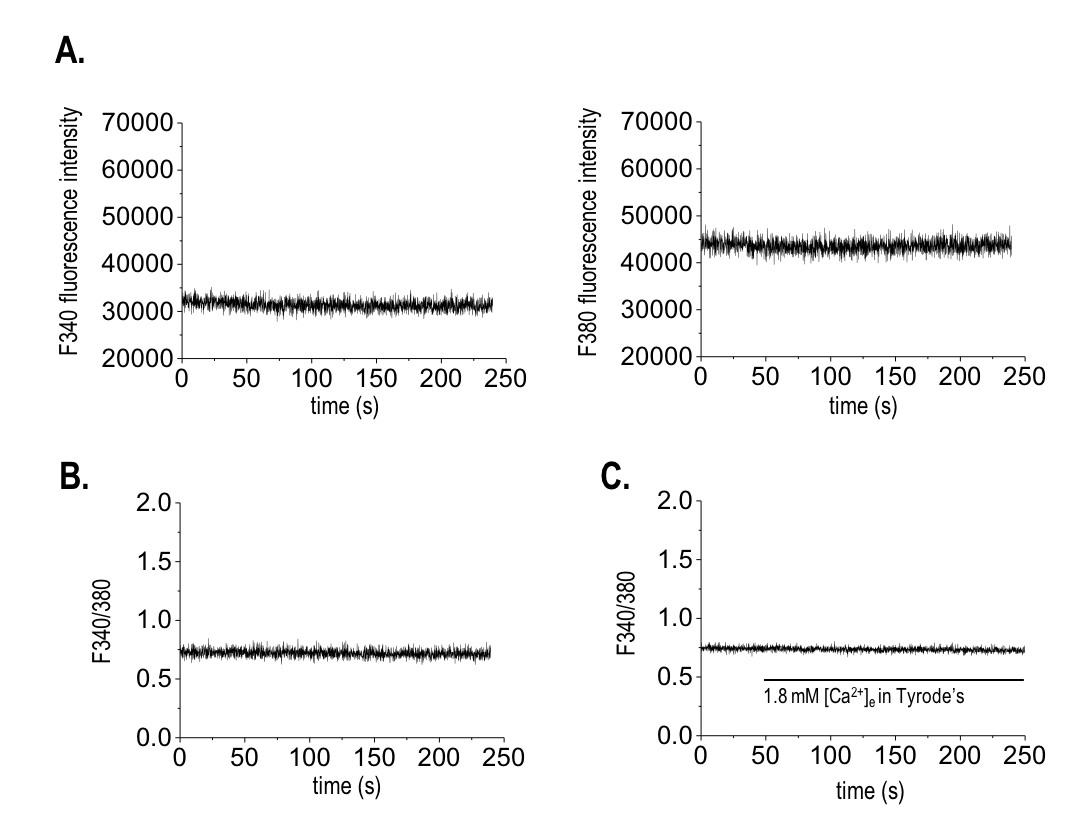


**Figure S7.** Fluorescent measurement of intracellular calcium concentration in Fura2-AM loaded 2- and 3-day-old cells of HD cultures under control conditions. Resting fluorescence (F) detected at 510 nm following excitation at 340 nm and 380 nm wavelength (**A**), and the ratio (F340/F380) of fluorescence intensities was calculated and plotted (**B**). Cells were kept in Tyrode’s standard salt solution during the measurement. F340/380 ratio in control conditions recorded on a representative 3-day-old cell in a HD culture (**C**). The cell was first kept in Tyrode’s solution, then, as indicated, the local perfusion with the same solution was turned on, causing no alterations in the detected fluorescence levels.

*10. Electrophysiology*

Application of 20 µM NMDA and 5 µM glycine or 300 µM NMDA and 10 µM glycine simultaneously in the extracellular solution had no significant effect on the amplitude of the measured current when the cell was held continuously at –60 mV (*Figure S8A–C*). To test the possible effect of the agonists, voltage ramp protocols were also applied with which any change of the reversal potential due to the activation of NMDARs could be detected. Application of the test substances, however, had no significant effect on the reversal potential of the measured current (*Figre S8D–F*).


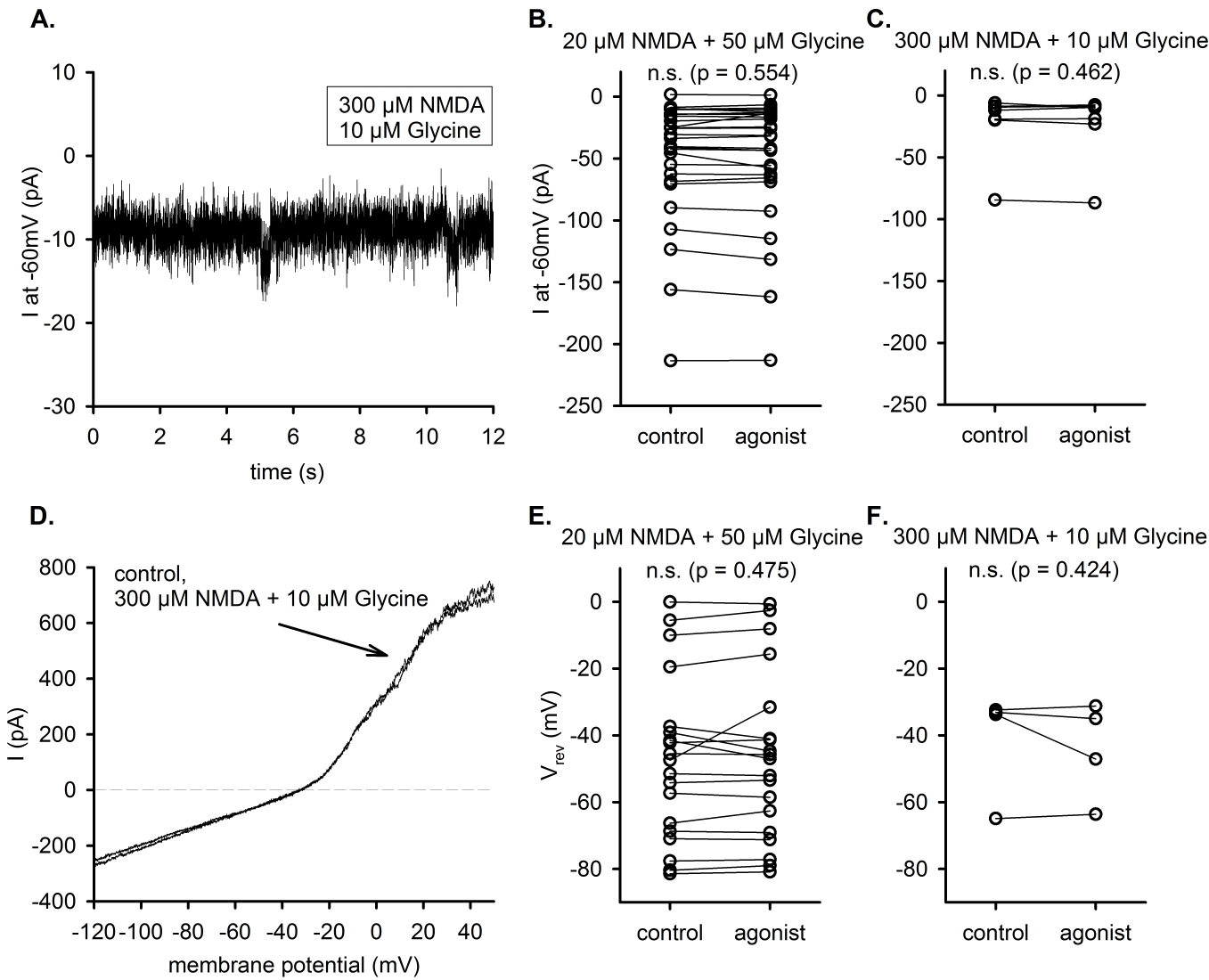


**Figure S8.** Whole-cell patch-clamp recordings on differentiating chondrocytes in voltage-clamp mode on culturing days
2 and 3. **A.** Whole-cell currents were recorded at a holding potential of –60 mV. Agonists were applied in the bath solution (indicated by box, typical record is shown). Extracellular application of (**B**) 20 µM NMDA and 5 µM glycine (*n* = 28) or
(**C**) 300 µM NMDA and 10 µM glycine (*n* = 7) had no significant effect on the measured current (n.s., paired Student’s *t*-test).
**D.** Voltage ramps were recorded from –120 mV to +50 mV every 15 s from a holding potential of –120 mV in the absence and in the presence of (**E**) 20 µM NMDA and 5 µM glycine (*n* = 19) or (**F**) 300 µM NMDA and 10 µM glycine (*n* = 4).
No significant effect on the reversal potential (V_rev_) of the current was detected (n.s., paired Student’s *t* -test). Typical records are shown. Data was collected in 23 independent experiments.

*11. Chondrogenic marker gene expression during and after transient NR1 gene silencing*

*
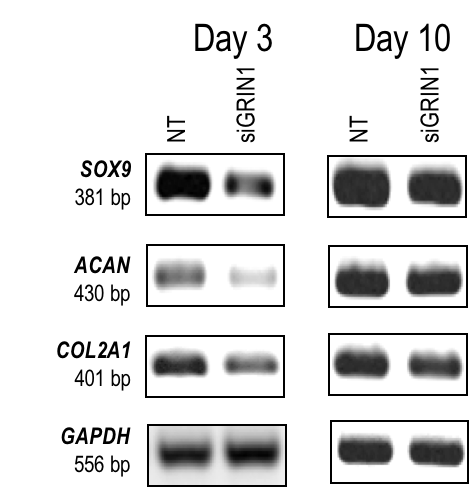
*

**Figure S9.** mRNA expression profile of chondrogenic marker genes in chondrifying chicken micromass cultures following transfection with non-targeting (NT) or *GRIN1*-specific (siNR1) siRNA as determined by conventional RT-PCR on culturing days 3 and 10. Representative images out of 3 independent experiments, each showing a similar expression profile.

*12. Calculations for estimating the Ca^2+^ current amplitude based on the parameters of Ca^2+^ transients obtained during fluorescent single cell Ca^2+^ imaging*

Assuming a d = 20 μm cell diameter and a spherical cell, the cell volume is

$V=4.2 \cdot{10}^{-12} L$.

The Ca^2+^ that needs to enter the cell to raise the concentration by 60 nM is

$$60 nmole\cdot L^{-1} \cdot4.2 \cdot{10}^{-12}=2.5 \cdot{10}^{-19} mole \cdot6 \cdot{10}^{23}=1.5 \cdot{10}^{5}$$

Ca^2+^ ions. These carry

$$1.5 \cdot{10}^{5} \cdot2 \cdot1.6 \cdot{10}^{-19} C=4.8 \cdot{10}^{-14} C$$

charge into the cell. If this charge enters in 1 s then it corresponds to

$$I=\frac{4.8 \cdot{10}^{-14}}{1 s}=4.8 \cdot{10}^{-14} A=0.048 pA$$

current. This represents an upper limit, since the cytoplasmic volume is likely to be much lower due to the nucleus and organelles, requiring fewer Ca^2+^ ions for the 60 nM signal. In addition, the entry of the calculated charge may take much longer than 1 s, which would further reduce the required current.
